# cGAS‐STING Pathway Activation and Systemic Anti‐Tumor Immunity Induction via Photodynamic Nanoparticles with Potent Toxic Platinum DNA Intercalator Against Uveal Melanoma

**DOI:** 10.1002/advs.202302895

**Published:** 2023-10-09

**Authors:** Hui Tao, Jia Tan, Hanchen Zhang, Hong Ren, Ziyi Cai, Hanhan Liu, Bingyu Wen, Jiaqi Du, Gaoyang Li, Shijie Chen, Haihua Xiao, Zhihong Deng

**Affiliations:** ^1^ Department of Ophthalmology The Third Xiangya Hospital Central South University Changsha Hunan 410013 P. R. China; ^2^ Eye Center of Xiangya Hospital Central South University Changsha Hunan 410008 P. R. China; ^3^ Hunan Key Laboratory of Ophthalmology and National Clinical Research Center for Geriatric Disorders, Xiangya Hospital, Central South University Changsha Hunan 410008 P. R. China; ^4^ Beijing National Laboratory for Molecular Sciences Laboratory of Polymer Physics and Chemistry Institute of Chemistry Chinese Academy of Sciences Beijing 100190 China; ^5^ University of Chinese Academy of Sciences Beijing 100049 China; ^6^ Department of Spine Surgery The Third Xiangya Hospital Central South University Changsha Hunan 410013 P. R. China

**Keywords:** 56MESS, cGAS‐STING, immunotherapy, nanoparticles, uveal melanoma

## Abstract

The cGAS‐STING pathway, as a vital innate immune signaling pathway, has attracted considerable attention in tumor immunotherapy research. However, STING agonists are generally incapable of targeting tumors, thus limiting their clinical applications. Here, a photodynamic polymer (P1) is designed to electrostatically couple with 56MESS–a cationic platinum (II) agent–to form NP^PDT^‐56MESS. The accumulation of NP^PDT^‐56MESS in the tumors increases the efficacy and decreases the systemic toxicity of the drugs. Moreover, NP^PDT^‐56MESS generates reactive oxygen species (ROS) under the excitation with an 808 nm laser, which then results in the disintegration of NP^PDT^‐56MESS. Indeed, the ROS and 56MESS act synergistically to damage DNA and mitochondria, leading to a surge of cytoplasmic double‐stranded DNA (dsDNA). This way, the cGAS‐STING pathway is activated to induce anti‐tumor immune responses and ultimately enhance anti‐cancer activity. Additionally, the administration of NP^PDT^‐56MESS to mice induces an immune memory effect, thus improving the survival rate of mice. Collectively, these findings indicate that NP^PDT^‐56MESS functions as a chemotherapeutic agent and cGAS‐STING pathway agonist, representing a combination chemotherapy and immunotherapy strategy that provides novel modalities for the treatment of uveal melanoma.

## Graphical abstract

To activate the systemic anti‐tumor immunity, photodynamic nanoparticles with potent Toxic platinum (NP^PDT^‐56MESS) are successfully prepared. NP^PDT^‐56MESS activates the cGAS‐STING pathway via ROS and 56MESS released upon irritated by a NIR laser (NP^PDT^‐56MESS +L). Furthermore, NP^PDT^‐56MESS + L eradicates tumors and dramatically inhibits the recurrence and metastasis of tumors in mice.

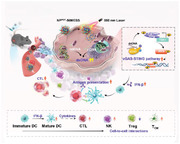

## 1 Introduction

Uveal melanoma (UM), as the most common primary intraocular malignancy, is prone to metastasis, resulting in a one‐year survival rate of < 50%.^[^ ^1^ ^]^ Although immunotherapy has the potential to improve patient survival,^[^ ^2^ ^]^ patients with UM exhibited poor responses to immunotherapy, e.g., immune checkpoint blockade (ICB).^[^ ^3^ ^]^ The main reason for this is the highly immunosuppressive microenvironment in UM tumors.^[^ ^1^, ^4^ ^]^ Moreover, low bioavailability and systemic toxicity of agents decreased the efficiency of immunotherapy.^[^ ^5^ ^]^ Therefore, optimized immunotherapy strategies are continued and need to be explored for uveal melanoma.

cGAS‐STING pathway is a natural immune signaling pathway, the activation of which can secrete the type I interferons (IFNs) to promote dendritic cell (DC) maturation, and subsequently increased immune cell infiltration into the tumor microenvironment (TME) (e.g., natural killer cells [NKs] and cytotoxic T cells [CTLs]), thus eliciting an anti‐tumor immune response in vivo.^[^ ^6^ ^]^ Therefore, immunotherapy based on activation of the cGAS‐STING pathway may prove effective in the treatment of UM. However, STING agonists, typically, do not target tumors and are prone to degradation in vivo, thus limiting their clinical application.^[^ ^7^ ^]^ As a number of novel strategies were applied to activate cGAS‐STING pathway,^[^ ^8^ ^]^ a system combination of immunotherapy and chemotherapeutic seems to be especially efficient.

Platinum drugs, such as cisplatin (Cis) and carboplatin, activate the cGAS‐STING signaling pathway to some extent via leaked double‐stranded DNA (dsDNA) into the cytoplasm.^[^ ^9^ ^]^ 56MESS ([5,6‐dimethyl‐1,10‐phenanthroline] [1S,2S‐diaminocyclohexane] platinum [II]) is also a platinum‐based chemotherapeutic agent with potent anti‐cancer activity.^[^ ^10^ ^]^ More specifically, 56MESS inserts itself directly into DNA, thereby damaging the structure of DNA, and causing nuclear DNA (nDNA) leakage into the cytoplasm.^[^ ^11^ ^]^ 56MESS also has the potential to cause mitochondrial perforation, leading to the release of mitochondrial DNA (mtDNA).^[^ ^12^ ^]^ Therefore, the dual action of 56MESS on nuclear DNA and mitochondria may cause a surge of dsDNA to rapidly reach the threshold level for activation of the cGAS‐STING pathway.^[^ ^6b^ ^]^ However, the therapeutic applications of 56MESS are hindered by its intense systemic toxicity^[^ ^11^, ^13^ ^]^; hence, a safe and efficient delivery system is needed to send 56MESS directly to tumors.

**Scheme 1:**
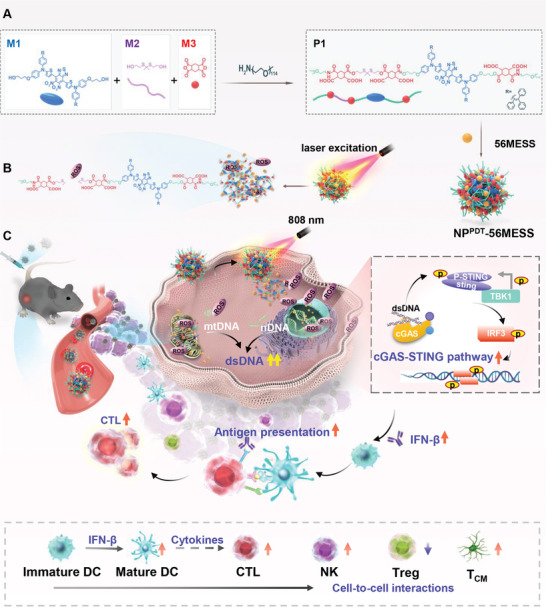
Schematic illustration of cGAS‐STING pathway activation and anti‐tumor immunity induction via NP^PDT^‐56MESS + L. A) Preparation of NP^PDT^‐56MESS via self‐assembly of P1 with 56MESS. B) NP^PDT^‐56MESS produces ROS and releases 56MESS upon excitation by a NIR 808 nm laser. C) NP^PDT^‐56MESS + L increases dsDNA in the cytoplasm, which activates the cGAS‐STING pathway. Subsequently, IFN‐β is released by tumor cells to promote DC maturation, inducing an anti‐tumor immune response in vivo.

Here, an amphiphilic polymer (P1) was synthesized via the condensation polymerization of a photodynamic monomer (M1), a thioketal‐containing monomer (M2), and anhydride monomers (M3), which was further end‐capped with methoxypolyethylene glycol (mPEG_5000_‐OH)^[^ ^14^ ^]^ (**Scheme** **1**A). M1 can be excited by an 808 nm laser to generate reactive oxygen species (ROS),^[^ ^15^ ^]^ which can immediately break the thiol‐ketal bonds in M2 and result in the rapid degradation of P1.^[^ ^16^ ^]^ Subsequently, P1, which contains pendant carboxylic acids, was used to encapsulate 56MESS via electrostatic interactions to form nanoparticles, designated NP^PDT^‐56MESS (Scheme 1B). Injection of NP^PDT^‐56MESS into tumor‐bearing mice with UM resulted in the accumulation of NP^PDT^‐56MESS at the tumor site. NP^PDT^‐56MESS may induce the production of a large amount of ROS following light irradiation with an 808 nm laser (NP^PDT^‐56MESS + L), which then disintegrates the nanoparticles and subsequently releases 56MESS. The ROS and 56MESS can severely damage the DNA and mitochondria and thus release dsDNA to activate cGAS‐STING pathway to induce the anti‐tumor effect (Scheme 1C). Hence, NP^PDT^‐56MESS + L not only kills cancer cells by ROS and 56MESS, but also effectively activates the cGAS‐STING pathway to elicit anti‐tumor immunity, representing a combined chemotherapy and immunotherapy strategy for the treatment of UM.

## 2 Results and Discussion

### 2.1 Preparation and Characterization of NP^PDT^‐56MESS

P1 is an amphiphilic polymer with hydrophilic mPEG and hydrophobic segments containing monomers M1, M2, and M3 (Scheme S1 and Figure S1, Supporting Information). The average molecular weight (Mw) of P1 was 16358 by gel permeation chromatography (GPC) (Table S2, Supporting Information). Moreover, the critical micelle concentration (CMC) for P1 was found to be 0.01 mg mL^−1^ (Figure S2, Supporting Information). Due to its amphiphilic nature, P1 self‐assembles in an aqueous solution to form nanoparticles, named NP^PDT^ (Scheme S2, Supporting Information). Additionally, P1 with pair‐wise carboxyl groups encapsulates cationic 56MESS into nanoparticles (NP^PDT^‐56MESS) via electrostatic interactions (**Scheme** **1**A).

**Figure 1:**
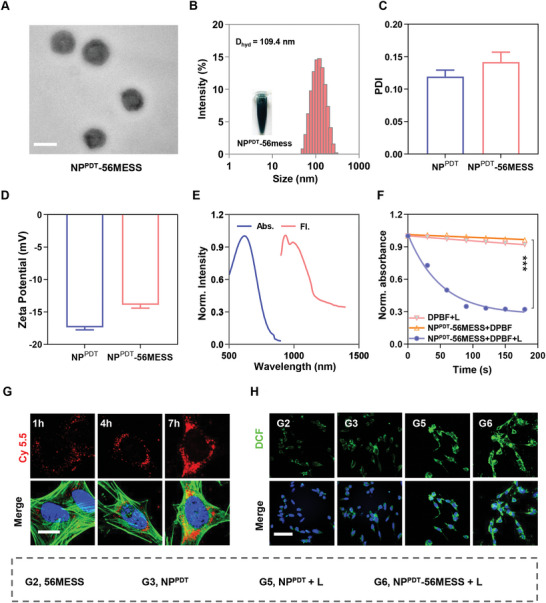
Characterization of NP^PDT^‐56MESS. A) Representative TEM image of NP^PDT^‐56MESS. Scale bar: 100 nm. B) The diameter of NP^PDT^‐56MESS by DLS. C,D) PDI and zeta potential of NP^PDT^ and NP^PDT^‐56MESS by DLS. E) Absorption and fluorescence emission spectra of NP^PDT^‐56MESS. F) ROS generation of NP^PDT^‐56MESS + L revealed by diphenylisobenzofuran (DPBF) absorbance change at 415 nm. G) Representative Confocal fluorescence microscopy (CLSM) images of intracellular uptake of Cy5.5‐labeled NP^PDT^‐56MESS at 1, 4, and 7 h. (Blue, DAPI; Green, Actin; Red, Cy 5.5)Scale bar: 20 µm. H) ROS generation of various treatments in the dark or upon light irradiation (808 nm, 1 W cm^−2^, 3 min) by CLSM. (Blue, DAPI; Green, DCF;) Scale bar: 60 µm.

Transmission electron microscopy (TEM) revealed that NP^PDT^‐56MESS has a uniform, spherical shape with an approximate diameter of 100 nm (**Figure** **1**A). Further characterization by dynamic light scattering (DLS) indicated that the average particle sizes of NP^PDT^ and NP^PDT^‐56MESS were 108.1 and 109.4 nm (Figure 1B; Figure S3A, Supporting Information), with polydispersity index (PDI) values of 0.12 and 0.14 (Figure 1C) and zeta potentials of −17.4 and −13.9 mV, respectively (Figure 1D). In addition, the particle sizes of NP^PDT^ and NP^PDT^‐56MESS remained relatively unchanged throughout four weeks of storage in phosphate‐buffered saline (PBS) (Figure S3B,C, Supporting Information), and remained almost constant within 5 days in DMEM containing 10% FBS (Figure S3D,F, Supporting Information), indicating that they both had good stability.

The photophysical properties of the nanoparticles were then investigated. The absorption spectrum of NP^PDT^‐56MESS ranged from 400–900 nm by UV–vis–NIR spectroscopy, with the absorption peak at 620 nm. Moreover, the emission spectrum of NP^PDT^‐56MESS was within the near‐infrared wavelength region (NIR II, 900–1700) with an emission peak at 931 nm under 808 nm laser excitation (Figure 1E). Notably, the absorption and emission spectra of NP^PDT^‐56MESS were relatively the same as those of P1,^[^ ^17^ ^]^ confirming the formation of NP^PDT^‐56MESS by P1.

Furthermore, the ability of NP^PDT^‐56MESS to generate ROS was explored. 1,3‐Diphenylisobenzofuran (DPBF) is a fluorescent probe that can be decomposed by ROS, resulting in a decrease in the OD value of DPBF at 415 nm. The results showed the OD values decreased by 50.00% upon irradiation of NP^PDT^‐56MESS with an 808 nm laser for 60 s and by 65.09% after 90 s (Figure 1F). Additionally, TEM results indicated that NP^PDT^‐56MESS was disintegrated into flocculent fragments following 180 s excitation (Figure S4A, Supporting Information). Moreover, 75.54% Pt content was released within 24 h upon irradiation of NP^PDT^‐56MESS while it remained intact in the dark (Figure S4B, Supporting Information). Taken together, these findings indicated that NP^PDT^‐56MESS + L rapidly produced a large amount of ROS to rapidly disintegrate the nanoparticles.

### 2.2 In Vitro Study of NP^PDT^‐56MESS

In order to observe how readily NP^PDT^‐56MESS is taken up, OCM‐1 cells were treated with NP^PDT^‐56MESS@Cy5.5 (NP^PDT^‐56MESS labeled by cy5.5) for different durations. Confocal fluorescence microscopy (CLSM) results showed a gradual increase in the fluorescence intensity of intracellular cy5.5 in a time‐dependent manner (Figure 1G; Figure S5, Supporting Information). Similarly, flow cytometric (FCM) findings indicated that the mean fluorescence intensity (MFI) had increased by ≈20‐fold following 7 h of treatment compared with that at 1 h (Figure S6, Supporting Information). These results suggested that NP^PDT^‐56MESS was successfully taken up by OCM‐1 cells and that the intracellular uptake process was time‐dependent.

Next, the ability of NP^PDT^ or NP^PDT^‐56MESS to generate ROS under the irradiation of 808 nm laser (NP^PDT^ + L or NP^PDT^‐56MESS + L) was investigated via DCFH‐DA probes by CLSM and FCM. CLSM observations revealed a marked increase in the fluorescence of DCF within OCM‐1 cells treated with NP^PDT^ + L or NP^PDT^‐56MESS + L (Figure 1H; Figure S7, Supporting Information). Meanwhile, the MFI of DCF in cells treated with NP^PDT^‐56MESS + L or NP^PDT^ + L was nearly 30‐fold higher than that in cells treated with either NP^PDT^‐56MESS or NP^PDT^ by FCM (Figure S8, Supporting Information). These results indicated that NP^PDT^ + L or NP^PDT^‐56MESS + L produced abundant ROS in cells.

### 2.3 Anti‐Tumor Effect Elicited by NP^PDT^‐56MESS + L In Vitro

**Figure 2:**
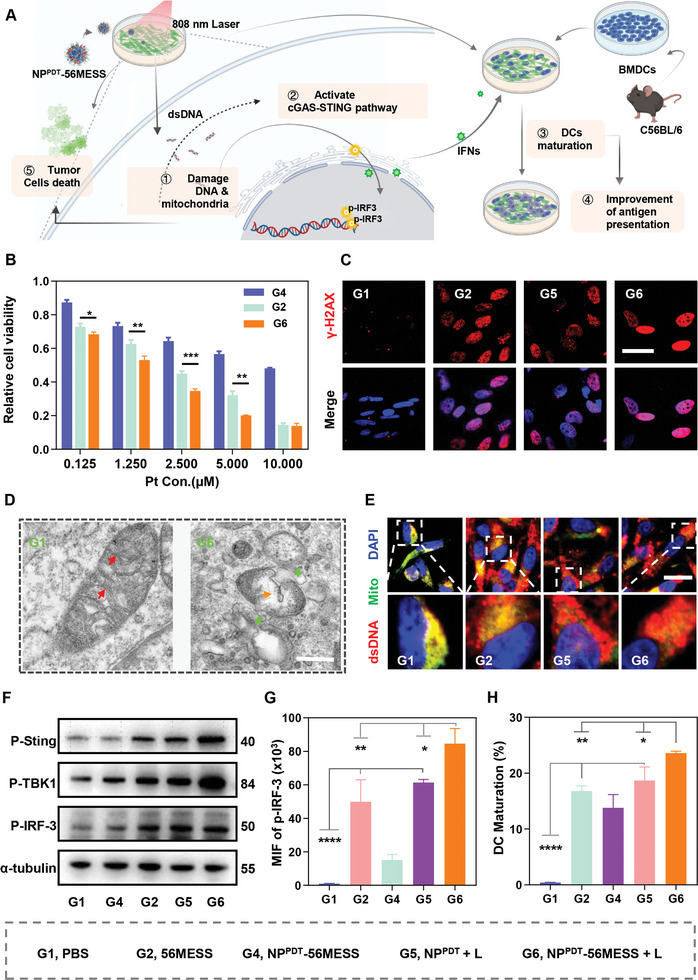
Anti‐tumor effect of NP^PDT^‐56MESS + L in vitro. A) Schematic illustration of cGAS‐STING pathway activation to induce an anti‐tumor effect in cells treated with NP^PDT^‐56MESS + L. (Green cells, tumor cells; Bule cells, BMDCs; Purple cells, mature DCs). B) Relative cell viability of OCM‐1 cells treated with various drugs for 24 h via MTT assay. C) Representative CLSM images of DNA damage marker γ‐H2AX in OCM‐1 cells treated with various drugs for 12 h. (Blue, DAPI; Red, γ‐H2AX). Scale bar: 40 µm. D) Representative TEM images of mitochondria in OCM‐1 cells treated with PBS or NP^PDT^‐56MESS + L for 12 h. (Red arrow, mitochondrial cristae; Orange arrow, mitochondrial vacuolation; Green arrow, rupture of mitochondrial membrane). Scale bar: 500 nm. E) Representative CLSM images of dsDNA in the cytoplasm of OCM‐1 cells after various treatments for 12 h (Blue, DAPI; Green, mitochondria; Red, dsDNA; the color of dsDNA in mitochondria is yellow while the color of the dsDNA outside of the mitochondria is red in the merged images). Scale bar: 20 µm. F) Expression of cGAS‐STING proteins in OCM‐1 cells after various treatments for 24 h by WB. α‐tubulin was used as the internal reference protein. G) FCM quantification of p‐IRF3 proteins in OCM‐1 cells after various treatments for 24 h. H) FCM quantification of mature bone‐marrow‐derived dendritic cells (BMDCs). Data are presented as mean ± SD. Statistical significance between every two groups was calculated via one‐way ANOVA. ^*^ *p* < 0.05, ^**^ *p* < 0.01, ^***^ *p* < 0.001, and ^****^ *p* < 0.0001.

The ROS produced by NP^PDT^‐56MESS + L would immediately degrade NP^PDT^ ‐56MESS and release 56MESS. Subsequently, ROS and 56MESS will damage cellular DNA and mitochondria to induce anti‐cancer effects in tumor cells (**Figure** **2**A). Results showed that the IC_50_ of NP^PDT^‐56MESS + L was 1.46 µm in OCM‐1 cells, while that of 56MESS was 2.23 µm (Figure 2B). Meanwhile, the maximum inhibition rate of NP^PDT^ + L‐treated OCM‐1 cells approached 30% (Figure S9A, Supporting Information). Similar results were observed in the B16‐F10 cells (Figure S9B,C, Supporting Information). These findings indicated that NP^PDT^ ‐56MESS + L exerted the most potent anti‐cancer activity, which may be due to synergistic effects between 56MESS and ROS.

Subsequently, the effect of NP^PDT^‐56MESS + L on cellular DNA was explored. γ‐H2AX is a marker of the DNA damage,^[^ ^16^, ^18^ ^]^ CLSM analysis revealed that the fluorescence of γ‐H2AX was the most intense within cells treated with NP^PDT^‐56MESS + L (Figure 2C; Figure S10A, Supporting Information), and the MFI of γ‐H2AX in cells treated with NP^PDT^‐56MESS + L was 5.71‐, 2.60‐, and 1.54‐fold higher than that in cells treated with PBS, NP^PDT^ + L, and 56MESS by FCM, respectively (Figure S10B, Supporting Information). Hence, NP^PDT^‐56MESS+ L induced remarkable DNA damage via a synergistic effect elicited by 56MESS and ROS.

Next, the effects of NP^PDT^‐56MESS + L on mitochondria were explored. Firstly, mitochondrial‐specific ROS was marked by the MitoSOX Red probe.^[^ ^19^ ^]^ The fluorescence of MitoSOX was particularly evident in cells treated with NP^PDT^‐56MESS + L (Figure S11, Supporting Information), indicating mitochondrial stress occurred in these cells. Mitochondrial dysfunction was further reflected by decreased ratios of JC‐1 aggregation/JC‐1 monomer.^[^ ^20^ ^]^ The ratios of JC‐1 aggregation/JC‐1 monomer in cells treated with NP^PDT^‐56MESS + L (10.55%) was significantly decreased when compared to that of cells treated with PBS (81.30%), indicating the prominent mitochondrial dysfunction occurred in NP^PDT^‐56MESS + L treated cells (Figure S12, Supporting Information). In addition, significant changes were observed in mitochondrial morphology and structure within cells treated with NP^PDT^‐56MESS + L by TEM, e.g., mitochondrial and swelling membrane rupture (Figure 2D). Therefore, NP^PDT^‐56MESS + L induced a significant damaging effect on mitochondria.

### 2.4 Activation of the cGAS‐STING Pathway by NP^PDT^‐56MESS + L In Vitro

As cyclic GMP–AMP synthase (cGAS) sensed the abnormal dsDNA within the cytoplasm, proteins like STING, TBK1, and IRF‐3 were successive phosphorylated to activate the cGAS‐STING pathway (Figure 2A).^[^ ^6a^ ^]^ The efficacy of NP^PDT^‐56MESS + L in activating this pathway was further evaluated.

First, mitochondria and dsDNA were stained with anti‐TOMM20 antibody and dsDNA marker, respectively.^[^ ^21^ ^]^ CLSM images showed a remarkable red fluorescence of dsDNA outside the mitochondria in cells treated with NP^PDT^‐56MESS + L (Figure 2E; Figure S13A, Supporting Information). Moreover, the cytosolic mtDNA increased by 3.45‐fold after the treatment of NP^PDT^‐56MESS + L by qPCR analysis (Figure S13B, Supporting Information). These results above indicated that NP^PDT^‐56MESS + L induced remarkable dsDNA accumulated in the cytoplasm. Second, western blotting (WB) analysis revealed that the expression levels of p‐STING, p‐TBK1, and p‐IRF‐3 were significantly increased in NP^PDT^‐56MESS + L‐treated cells (Figure 2F; Figures S14 and S15, Supporting Information). Third, the MFI of p‐IRF‐3 in cells treated with NP^PDT^‐56MESS + L was 1.38‐, 5.58‐, and 84.61‐fold higher than that of cells treated with 56MESS, NP^PDT^ + L, and PBS by FCM, respectively (Figure 2G). Taken together, these results indicated that NP^PDT^‐56MESS + L resulted in the accumulation of dsDNA in the cytoplasm and activated the cGAS‐STING pathway via cascade activation of target proteins.

Activation of the cGAS‐STING pathway leads to an increase in the production of type I IFNs and pro‐inflammatory cytokines, which then induce an anti‐tumor immune response, including the maturation of DCs (Figure 2A).^[^ ^22^ ^]^ ELISA results showed that the abundance of IFN‐β and IL‐6 were 4.90 and 4.93 times higher in the supernatants of OCM‐1 cells treated with NP^PDT^‐56MESS + L when compared with those of cells treated with PBS (Figure S16, Supporting Information). Subsequently, bone‐marrow‐derived dendritic cells (BMDCs) were co‐incubated with B16‐F10 cells that had been treated with the different study drugs. FCM analysis showed that the proportion of mature DCs (CD80^+^CD86^+^) was 1.40, 1.71, and 58.51 times higher in cells treated with NP^PDT^‐56MESS + L than in cells treated with 56MESS, NP^PDT^ + L, or PBS, respectively (Figure 2H; Figure S17, Supporting Information). Hence, NP^PDT^‐56MESS + L exhibited the strongest potential of inducing anti‐tumor immunity.

### 2.5 Metabolomic Analysis of OCM‐1 Cells Treated with NP^PDT^‐56MESS + L

**Figure 3:**
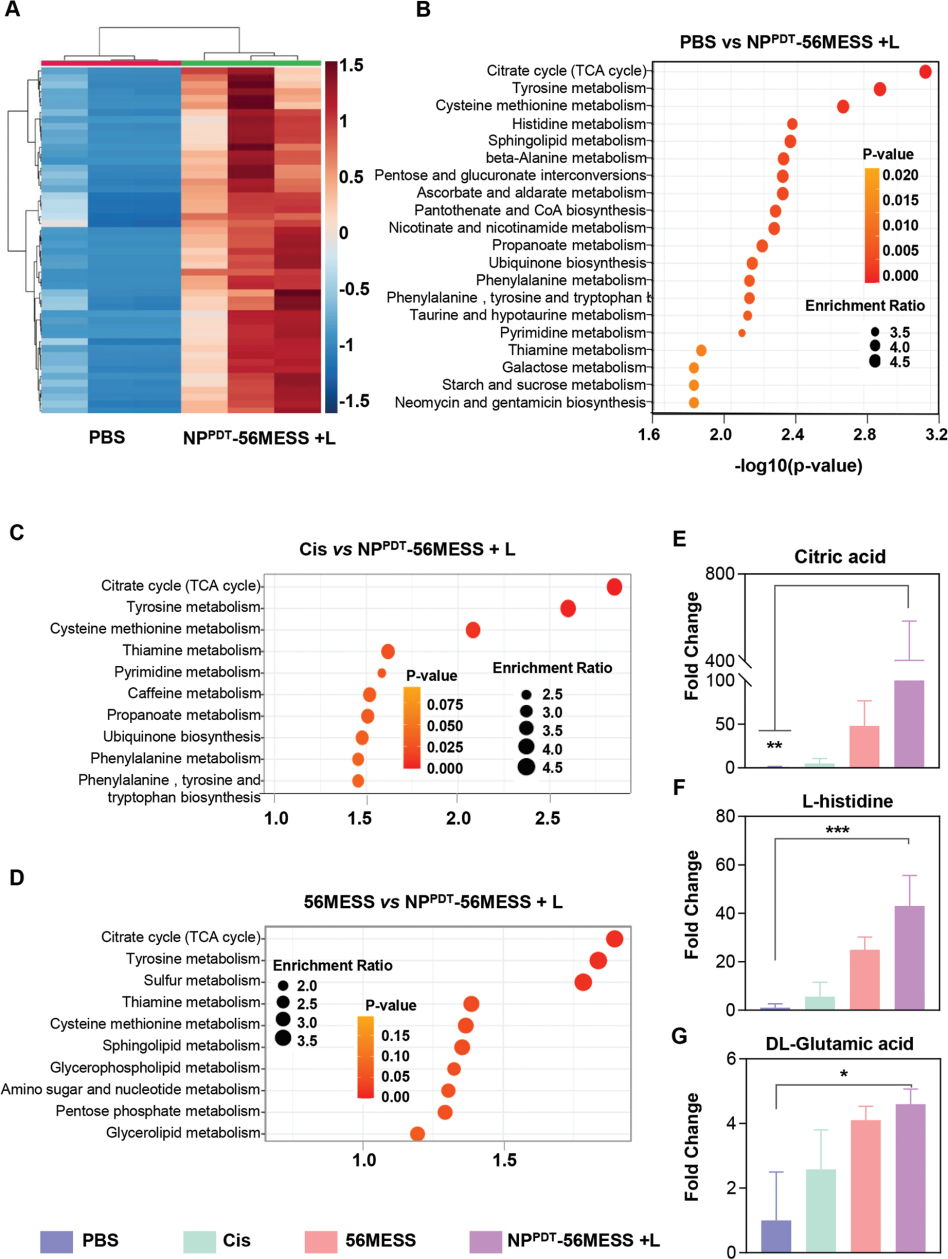
Metabolomic analysis of OCM‐1 cells treated with NP^PDT^‐56MESS + L. A) A heat map based on the different metabolites between the cells treated with PBS and NP^PDT^‐56MESS + L. B) Dot plot depicting the differential KEGG pathways between cells treated with PBS and NP^PDT^‐56MESS + L, C) Cis and NP^PDT^‐56MESS + L, D) 56MESS and NP^PDT^‐56MESS + L. Dot size corresponds to the enrichment ratio; dot color corresponds to the *p*‐value. E–G) Fold changes of typical metabolites such as Citric acid, L‐histidine, and DL‐Glutamine in different groups. *n* = 3. Data are presented as mean ± SD. Statistical significance between every two groups was calculated via one‐way ANOVA. ^*^ *p* < 0.05, ^**^ *p* < 0.01, and ^***^ *p* < 0.001.

To further investigate the effects of NP^PDT^‐56MESS + L on tumor cells, metabolites in OCM‐1 cells treated with PBS, Cis, 56MESS, or NP^PDT^‐56MESS + L were analyzed by liquid chromatography‐mass spectrometry (LC‐MC/MS). First, principal component analysis (PCA) revealed that intracellular metabolites were significantly separated in cells treated with different drugs, with PC1 and PC2 values of 71.6% and 14.5%, respectively (Figure S18, Supporting Information). Second, different metabolites appeared in the heat map, indicating different effects elicited by the various treatments (**Figure** **3**A; Figure S19, Supporting Information). Third, the Kyoto Encyclopedia of Genes and Genomes (KEGG) pathway enrichment analysis revealed that the metabolites related to the tricarboxylic acid (TCA) cycle were significantly enriched in cells treated with NP^PDT^‐56MESS + L compared to cells treated with PBS, cisplatin, or 56MESS (Figure 3B–D), indicating significant dysfunction occurred on mitochondria in NP^PDT^‐56MESS + L treated‐cells. Furthermore, purine or pyrimidine metabolism was enriched in cells treated with 56MESS or NP^PDT^‐56MESS + L (Figure 3B,C; Figure S20, Supporting Information), highlighting the presence of DNA damage within these cells.^[^ ^23^ ^]^ In addition, the levels of citric acid, L‐Aspartic acid, and D/L‐glutamic acid in cells treated with NP^PDT^‐56MESS + L were ≈406.8, 3687.5, and 5.0 times higher than those in cells treated with PBS (Figure 3E–G), further suggesting that the impaired energy metabolism and dysfunction of nucleotide synthesis in cells treated with NP^PDT^‐56MESS + L.

### 2.6 Biosafety and Bio‐Distribution of NP^PDT^‐56MESS In Vivo

Biosafety was evaluated before the application of NP^PDT^‐56MESS for treatment in vivo. First, PBS, NP^PDT^, 56MESS, and NP^PDT^‐56MESS were administered to healthy KM mice via the tail vein, respectively. (Figure S21, Supporting Information). The results showed that the body weight fluctuated by < 10% following administration of the NP^PDT^‐56MESS or PBS (Figure S22, Supporting Information), indicating that the systemic toxicity of NP^PDT^‐56MESS at this dose was negligible. Furthermore, hematoxylin‐eosin (H&E) staining revealed no significant damage occurred in mice treated with NP^PDT^‐56MESS (Figure S23, Supporting Information). Additionally, the serum biochemical indices of mice treated with NP^PDT^‐56MESS did not significantly differ from those of PBS‐treated mice, while the levels of AST and ALT were significantly increased in mice treated with 56MESS (Figure S24A–I, Supporting Information). Therefore, these results indicated that NP^PDT^‐56MESS exhibited good biosafety in vivo.

**Figure 4:**
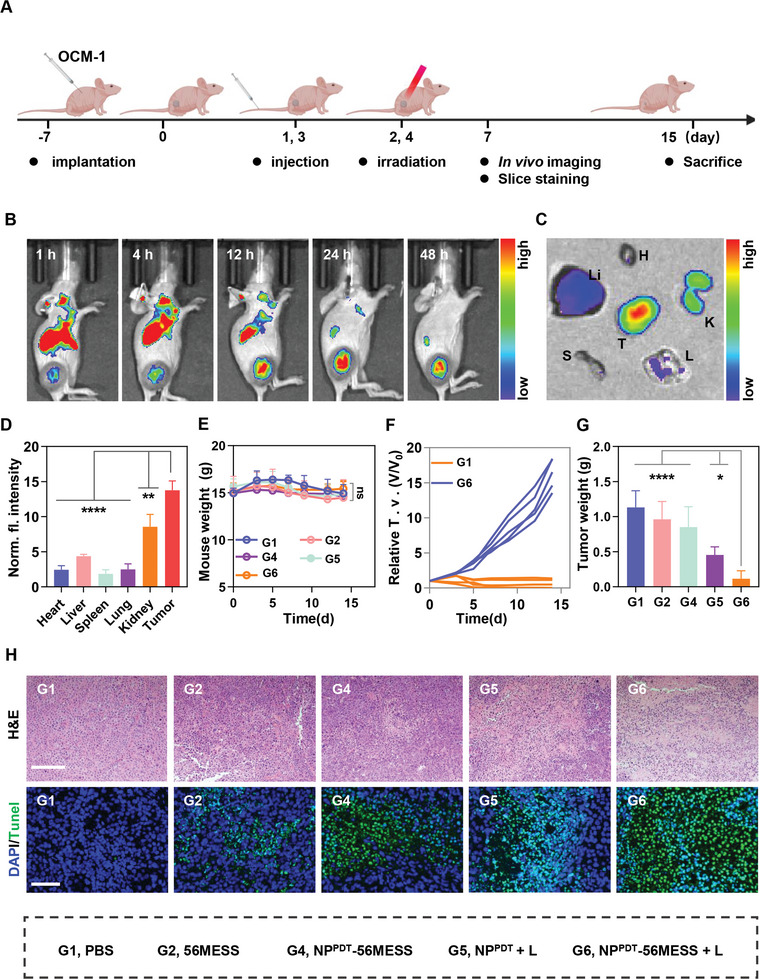
Biodistribution of NP^PDT^‐56MESS and anti‐tumor effect of the NP^PDT^‐56MESS + L in vivo. A) Schematic illustration of the in vivo study. B,C) Biodistribution of Cy7.5‐labeled NP^PDT^‐56MESS in OCM‐1 bearing mice via fluorescence imaging in vivo and in vitro tissues. T, H, Li, S, L, and K represent tumor, heart, liver, spleen, lung, and kidney, respectively. D) MFI of Cy7.5‐labeled NP^PDT^‐56MESS in major tissues at 48 h after intravenous injection. E–G) Body weight changes, tumor growth inhibition curves, and tumor weight of mice treated with various drugs (Pt at 0.4 mg kg^−1^, *n* = 5). H) H&E and TUNEL staining of tumor tissues in mice treated with various agents. Scale bar: 200 µm. Data are presented as mean ± SD. Statistical significance between every two groups was calculated via one‐way ANOVA. ^*^ *p* < 0.05 and ^****^ *p* < 0.0001.

Subsequently, the biodistribution of NP^PDT^‐56MESS was assessed in a subcutaneous model of BALB/c nude mice (**Figure** **4**A). First, mice were injected with NP^PDT^‐56MESS@Cy7.5 (NP^PDT^‐56MESS labeled Cy7.5) via the caudal vein and monitored with an *ex vivo* imaging System (IVIS) via fluorescence imaging. The fluorescence intensity of tumors continuously increased from 1 to 24 h, with a peak of 8.823 × 10^9^ p s^−1^ cm^−2^ S^−1^r^−1^ at 24 h. Thereafter, it gradually decreased to 8.08 × 10^9^ p^−1^ s^−1^ cm^−2^ S^−1^r^−1^ at 48 h (Figure 4B; Figure S25, Supporting Information). However, the fluorescence intensity in tumors remained 5.67, 3.15, 7.46, 5.53, and 1.61 times higher than those in the heart, liver, spleen, lung, and kidney at 48 h, respectively (Figure 4C,D). These results suggested that NP^PDT^‐56MESS targeted tumors and accumulated at tumors for extended periods of time.

### 2.7 Anti‐Tumor Effect of the NP^PDT^‐56MESS + L In Vivo

Next, the anti‐tumor effects of NP^PDT^‐56MESS + L were evaluated in the BALB/c nude mice bearing OCM‐1 tumors (Figure 4A). Firstly, no significant difference was observed in the weight among mice treated with different drugs (Figure 4E). Secondly, NP^PDT^‐56MESS + L treated mice had the smallest tumors on day 15 (Figure 4F,G; Figure S26, Supporting Information), e.g., the tumor weight in mice treated NP^PDT^‐56MESS + L was only 1/10 of that in mice treated with PBS. In addition, H&E staining showed remarkable nuclear fragmentation and nucleolytic in tumors of mice treated with NP^PDT^‐56MESS + L (Figure 4H, top panel). Similarly, significant DNA damage was observed in tumors of mice treated with NP^PDT^‐56MESS + L by TUNEL staining (Figure 4H, lower panel). These results suggested that NP^PDT^‐56MESS + L exhibited a remarkable tumor‐suppressive effect in vivo.

### 2.8 NP^PDT^‐56MESS +L Activates the cGAS‐STING Pathway to Enhance In Vivo Anti‐Tumor Effects

**Figure 5:**
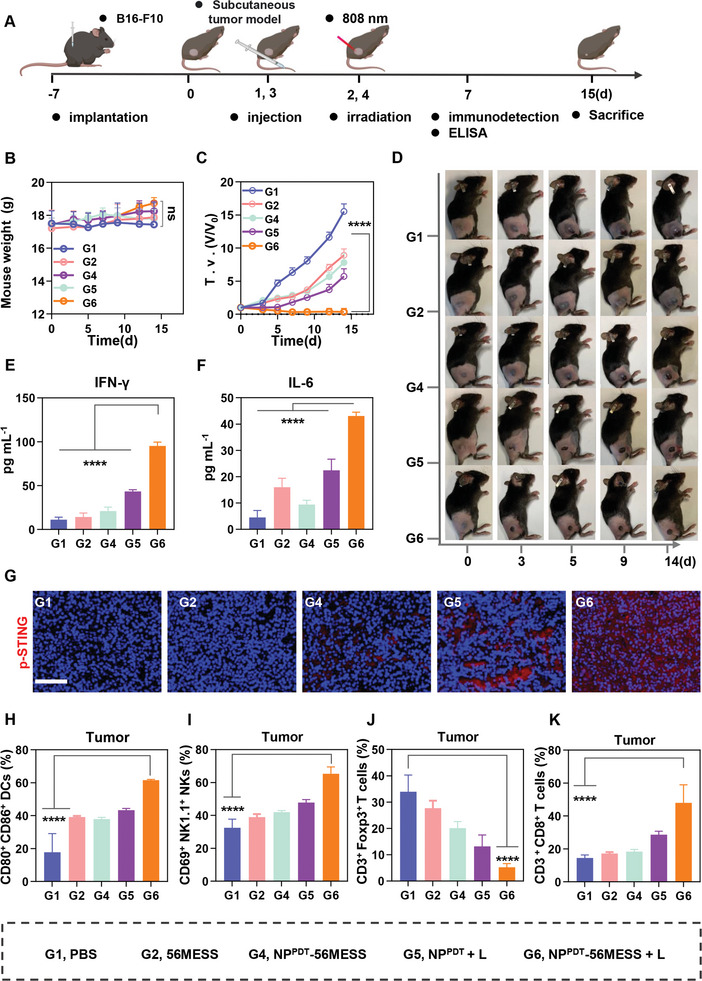
NP^PDT^‐56MESS + L activates the cGAS‐STING pathway to induce anti‐tumor effects in vivo. A) Schematic illustration of treatment schedules in vivo. B,C) Body weight changes and tumor growth inhibition curves of mice treated with various drugs (Pt at 0.4 mg kg^−1^, *n* = 5). D) Representative images of mice after treatment at various time points. E,F) The concentration of IFN‐γ and IL‐6 in mouse serum following various treatments. G) Immunofluorescence imaging of p‐STING in B16‐F10 tumors after different treatments (Blue, DAPI; Red, p‐STING). Scale bar: 50 µm. H–K) The proportions of mature DCs (CD80^+^CD86^+^), NKs (CD69^+^ NK1.1^+^), Tregs (CD4^+^Foxp3^+^), and CD8^+^ T cell (CD3^+^CD8^+^) populations within tumors. Data are presented as mean ± SD. Statistical significances between all groups were calculated via an unpaired two‐sided *t*‐test in (B and C), and one‐way ANOVA in (E, F, and H–K). ^****^ *p* < 0.0001.

NP^PDT^‐56MESS + L was found to activate the cGAS‐STING pathway in vitro. Next, the effect of NP^PDT^‐56MESS + L to induce anti‐tumor immunity was evaluated in vivo. To this end, we constructed the B16‐F10 tumor‐bearing C57BL/6 mouse model (**Figure** **5**A).

First, the activation of the cGAS‐STING pathway was investigated in vivo. No significant differences were observed in body weight between the mice treated with various agents (Figure 5B; Figure S27, Supporting Information), and tumors in mice treated with NP^PDT^‐56MESS + L had ceased growing or even dissipated by day 15 (Figure 5C,D; Figure S27, Supporting Information). Additionally, the levels of IFN‐γ, IL‐6, and IFN‐βwere 8.45, 9.53, and 4.42 times higher in the peripheral blood of mice treated with NP^PDT^‐56MESS + L than those of mice treated with PBS (Figure 5E,F; Figure S28, Supporting Information). Moreover, the fluorescence of p‐STING was especially noticeable in tumors of mice treated with NP^PDT^‐56MESS + L (Figure 5G). These results above suggested that NP^PDT^‐56MESS + L efficiently activated the cGAS‐STING pathway in vivo.

Next, we investigated the anti‐tumor immune response in vivo. FCM results showed that the relative proportion of mature DCs (CD80^+^CD86^+^) in tumor tissues of mice treated with NP^PDT^‐56MESS + L (61.57%) was higher than that of mice treated with PBS (17.80%) by 43.76% (Figure 5H; Figure S29, Supporting Information). Furthermore, the proportion of mature DCs within tumor‐draining lymph nodes (TDLNs) of mice treated with NP^PDT^‐56MESS + L (41.00%) was 1.90 times higher than that in mice treated with PBS (21.63%; Figure S30, Supporting Information). Collectively, these results indicated that NP^PDT^‐56MESS + L induced DC maturation in vivo.

Mature DCs, as the most potent antigen‐presenting cells, interact with myriad immune cells, resulting in the activation of NKs, downregulation of Tregs, and differentiation of naive T cells into CD8^+^ T cells.^[^ ^24^ ^]^ FCM analysis showed that: i) the proportion of NKs (CD69^+^NK1.1^+^) infiltrated in the tumor tissues of mice treated with NP^PDT^‐56MESS + L was 65.36%, representing a 32.87% increase compared with that in mice treated with PBS (32.50%) (Figure 5I; Figure S31, Supporting Information); ii) the proportion of Tregs (5.26%) in the tumor tissues of the mice treated with NP^PDT^‐56MESS + L was only 15.51% of that in mice treated with PBS (33.93%); Figure 5J and Figure S32 (Supporting Information); iii) The proportion of CD8^+^ T cells infiltrated in the tumor tissues of mice treated with NP^PDT^‐56MESS + L was 47.97%, which was 33.50% higher than that of mice treated with PBS (14.47%; Figure 5K; Figure S33, Supporting Information); iv) the proportion of infiltrated CD8^+^ T cells in the spleen of mice treated with NP^PDT^‐56MESS + L was 1.18 times higher than that of mice treated with PBS (Figure S34, Supporting Information). In addition, in order to visualize the infiltration of immune cells into tumor tissues, immunofluorescence staining was performed, revealing that the fluorescence intensity of CD8^+^ T cells was the highest in the tumor tissues of mice treated with NP^PDT^‐56MESS + L (Figure S35, Supporting Information). These results suggested that NP^PDT^‐56MESS + L effectively promoted the infiltration of anti‐tumor immune cells via activation of the cGAS‐STING pathway, thereby inducing a powerful anti‐tumor immune response in vivo.

### 2.9 NP^PDT^‐56MESS + L Protects Against Tumor Recurrence and Metastasis by Inducing Systemic Immunity

**Figure 6:**
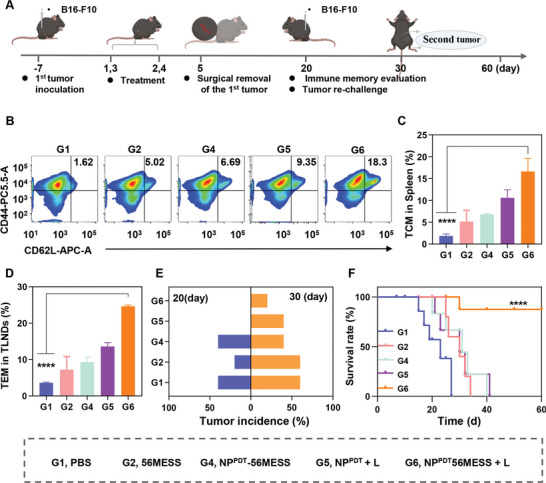
NP^PDT^‐56MESS + L protects against tumor recurrence or metastasis by inducing systemic immunity. A) Schematic illustration of treatment schedules in vivo. B,C) Representative FCM analysis images and populations of T_CM_ (CD44^+^ CD62L^+^) in the spleen. D) Populations of T_CM_ (CD44^+^ CD62L^+^) in TLNDs. E) Tumor incidence after resection of the primary tumor (*n* = 8 mice per group). F) Survival analysis of mice up to day 60. Data are presented as mean ± SD. Statistical significance between all groups was calculated via one‐way ANOVA in (C and D), and unpaired two‐sided *t*‐test in F. ^****^ *p* < 0.0001.

Mature DCs, NKs, and CD8^+^ T cells can further interact with each other or other immune cells, e.g., naïve T cells, which contributes to the development of systemic anti‐tumor immunity.^[^ ^25^ ^]^ Differentiated from naive T cells, memory T cells serve as the key components of the systemic anti‐tumor immune response, preventing tumor recurrence and metastasis.^[^ ^26^ ^]^ Hence, we further investigated the long‐term effects of NP^PDT^‐56MESS + L in the B16‐F10 tumor‐bearing C57BL/6 mouse model according to the workflow presented in **Figure** **6**A.^[^ ^27^ ^]^ FCM analysis revealed that the proportion of central memory T cell (*T*_CM_; CD44^+^CD62L^+^) infiltrated in the spleen of mice treated with NP^PDT^‐56MESS + L was 16.63%, which was 8.91 times higher than that of mice treated with PBS (1.87%); (Figure 6B,C; Figure S36, Supporting Information). Furthermore, the proportion of *T*_CM_ (CD44^+^ CD62L^+^) infiltrated in TDLNs of mice treated with NP^PDT^‐56MESS + L was 24.63%, which was 6.84 times higher than that of mice treated with PBS (3.60%; Figure 6D; Figure S37, Supporting Information). Meanwhile, no signs of recurring primary tumors were observed in mice treated with NP^PDT^‐56MESS + L, whereas the recurrence rate reached 40% in mice treated with PBS on day 20 (Figure 6E). As the rechallenge tumor model was established via subcutaneous injection of B16‐F10 cells into the opposite flank of mice, the incidence rate of developing a second tumor in mice treated with NP^PDT^‐56MESS + L was 20% by day 30 and 60% in mice treated with PBS (Figure 6E). Moreover, mice treated with NP^PDT^‐56MESS + L had the highest survival rate (80%), even at day 60 (Figure 6F). Taken together, NP^PDT^‐56MESS + L induced the systemic anti‐tumor immune effect to inhibit the recurrence and metastasis of B16‐F10 tumors and prolong the survival of mice.

## 3 Conclusion

In this study, nanoparticles named NP^PDT^‐56MESS were synthesized, which were excited by an 808 nm laser to generate ROS and subsequently release 56MESS. In vitro, the ROS and 56MESS synergistically damaged cellular DNA and mitochondria, resulting in a significant increase in cytoplasmic dsDNA, which subsequently activated the cGAS‐STING pathway. In vivo, NP^PDT^‐56MESS selectively accumulated at the tumor sites of mice with reduced systemic toxicity. NP^PDT^‐56MESS + L further induced the anti‐tumor immune responses to enhance the anti‐tumor effect via activating the cGAS‐STING pathway. NP^PDT^‐56MESS + L also induced systemic anti‐tumor immune memory in mice by increasing the proportion of infiltrating *T*_CM_ cells in the spleen, effectively inhibiting the recurrence and metastasis of melanoma. Importantly, the survival rate of mice treated with NP^PDT^‐56MESS + L remained as high as 80% even at day 60. Hence, NP^PDT^‐56MESS + L is not only an effective chemotherapeutic agent, but also a “STING agonist” capable of inducing anti‐tumor immunity. Taken together, this study provides an effective chemotherapy and immunotherapy combinatorial strategy for the treatment of UM, which has great potential for clinical application.

## Conflict of Interest

The authors declare no conflict of interest.

## Author Contributions

H.T. and J.T. contributed equally to this work. The manuscript was written through the contributions of all authors. All authors have given approval to the final version of the manuscript.

## Supporting information

Supporting Information

## Data Availability

The data that support the findings of this study are available from the corresponding author upon reasonable request.
