# Supplementary material for: cGAS‐STING Pathway Activation and Systemic Anti‐Tumor Immunity Induction via Photodynamic Nanoparticles with Potent Toxic Platinum DNA Intercalator Against Uveal Melanoma
Source: Adv Sci (Weinh). 2023 Oct 9;10(33):2302895. doi: 10.1002/advs.202302895 (PMC10667795; doi:10.1002/advs.202302895)
Supplement: Supplementary file 1 — Supporting Information [file ADVS-10-2302895-s001.pdf]

ADVANCED SCIENCE
Open Access

Supporting Information

for *Adv. Sci.*, DOI 10.1002/advs.202302895

cGAS-STING Pathway Activation and Systemic Anti-Tumor Immunity Induction via Photodynamic Nanoparticles with Potent Toxic Platinum DNA Intercalator Against Uveal Melanoma

*Hui Tao, Jia Tan, Hanchen Zhang, Hong Ren, Ziyi Cai, Hanhan Liu, Bingyu Wen, Jiaqi Du, Gaoyang Li, Shijie Chen, Haihua Xiao and Zhihong Deng**

Supporting Information

cGAS-STING Pathway Activation and Systemic Anti-tumor Immunity Induction *via* Photodynamic Nanoparticles with Potent Toxic Platinum DNA Intercalator against Uveal Melanoma

Hui Tao[a,‡], *Jia Tan*[b,c,‡], *Hanchen Zhang*[d,e], *Hong Ren*[a], *Ziyi Cai*[a], *Hanhan Liu*[a], *Bingyu Wen*[a], *Jiaqi Du*[a], *Gaoyang Li*[a], *Shijie Chen*[f], *Haihua Xiao*[d,e], *Zhihong Deng*[a,]*

[a] Department of Ophthalmology, The Third Xiangya Hospital, Central South University, Changsha, Hunan, 410013, P. R. China.

[b] Eye Center of Xiangya Hospital, Central South University, Changsha, Hunan 410008, P. R. China;

[c] Hunan Key Laboratory of Ophthalmology, Changsha, Hunan 410008, P. R. China.

[d] Beijing National Laboratory for Molecular Sciences, Laboratory of Polymer Physics and Chemistry, Institute of Chemistry, Chinese Academy of Sciences, Beijing 100190, China.

[e] University of Chinese Academy of Sciences, Beijing 100049, China.

[f] Department of Spine Surgery, The Third Xiangya Hospital, Central South University, Changsha, Hunan, 410013, P. R. China.

TABLE OF CONTENTS

1. Supplemental materials and methods 1
Scheme S1 8
Figure S1 9
Scheme S2 10
Figure S2 11
Figure S3 12
Figure S4 13
Figure S5 14
Figure S6 15
Figure S7 16
Figure S8 17
Figure S9 18
Figure S10 19
Figure S11 20
Figure S12 21
Figure S13 22
Figure S14 23
Figure S15 24
Figure S16 25
Figure S17 26
Figure S18 27
Figure S19 28
Figure S20 29
Figure S21 30
Figure S22 31
Figure S23 32
Figure S24 33
Figure S25 34
Figure S26 35
Figure S27 36
Figure S28 37
Figure S29 38

Figure S30 39
Figure S31 40
Figure S32 41
Figure S33 42
Figure S34 43
Figure S35 44
Figure S36 45
Figure S37 46
Table S1 47
Table S2 48
Table S3 49

1. Supplemental materials and methods

1.1. General measurements

The morphology and size were measured by transmission electron microscope (TEM, Hitachi HT 7700, Japan). Dynamic light scattering (DLS) was conducted by Malvern Zetasizer NanoZS90 (Malvern Instruments, Malvern, UK). Inductively Coupled Plasma (ICP) analysis was conducted by using Inductively Coupled Plasma Optical Emission Spectrometer (Agilent technologies 7700 series, U.S.A.). The absorbance spectra and fluorescence spectra were measured using a UV-VIS Spectrophotometer (UV-2600, Shimadzu, Japan) and fluorescence spectrometer (FLS980), respectively. Cell imaging was conducted byConfocal fluorescence microscopy (CLSM) (LSM-800, ZEISS, Germany) . The MTT assay and DPBF assay was conducted using a Microplate reader (SpectraMax, USA). *In vivo* imaging was conducted by an *In Vivo* Imaging System (IVIS, Perkin Elmer, USA). Flow cytometry (FCM) was done with a CytoFLEX Flow Cytometry (Beckman Coulter, USA). q-PCR was was conducted using a Real-Time Fluorescence PCR System (QuantStudio™ 7 Flex, Thermo Scientific™, USA).

1.2. Materials and agents.

3,3'-(((((5,6-dinitrobenzo[c][1,2,5]thiadiazole-4,7-diyl)bis(thiophene-5,2-diyl))bis((4-(1,2,2-triphenylvinyl)phenyl)azanediyl))bis(4,1-phenylene))bis(oxy))bis(propan-1-ol) ($C_{84}H_{64}N_6O_8S_3$, M_1) was synthesized as previously described. Methoxypolyethylene glycol ($mPEG_{5000}$-OH), 5,6-Dimethyl-1,10-phenanthroline , (1S,2S)-Cyclohexane-1,2-diamine, Potassium platinochloride(K_2PtCl_4) and 1,3-diphenylisobenzofurane (DPBF) were purchased from Aladdin (Shanghai, China). Other reagents were purchased from Energy Chemical *Co., Ltd.* (Shanghai, China). All chemicals were obtained from commercial sources and used without further purification unless otherwise noted.

Cell culture vessels were purchased from Corning (Corning, NY, USA). Dulbecco's modified Eagle's medium (DMEM) with 4.5 g glucose, trypsin-EDTA (0.25%), penicillin/streptomycin (P/S) and fetal bovine serum (FBS) were purchased from Gibco (Gran Island, NY, USA). 3-(4,5-dimethylthiazol-2-yl)-2,5-diphenyltetrazolium bromide (MTT) were purchased from Aladdin (Shanghai, China). Antibodies used for flow cytometry analysis and immunostaining were listed in the supplementary Table S1.

1.3. Methods

1.3.1. Synthesis of photodynamic and ROS sensitive polymer 1 (P1)

The dihydroxy photosensitizer was synthesized as previously described. [1] To a solution of above dihydroxy photosensitizer monomer (M_1) (20.0 mg, 0.014 mmol) and reactive oxygen species (ROS) sensitive monomer (M_2) (2,2'-(propane-2,2-diylbis(sulfanediyl)) bis (ehan-1-ol)

(11.0 mg, 0.056 mmol) in anhydrous DMF (5 mL) was quickly added 1,2,4,5-cyclohexanetetracarboxylic dianhydride (M_3) (16.6 mg, 0.077 mmol). After magnetic stirring for 24 h at room temperature, Methoxypolyethylene glycol ($mPEG_{5000}$-OH) (0.0074 mmol, 37 mg) was added to the reaction mixture. After magnetic stirring for another 12 h at 50 ℃, the mixture was added into 15 mL of deionized water under sonication, followed by dialysis in a dialysis bag (MWCO: 8000-14000 Da). After 72 h, the solution was freeze-dried under reduced pressure to obtained 22 mg polymer (P1).

1.3.2. Synthesis of 56MESS

The 56MESS was synthesized as previously described [2].

Preparation of cis-[$Pt(DMSO)_2Cl_2$]. DMSO (234 mg, 3.0 mmol, 3.0 eq), dissolved in water, was mixed with a filtered aqueous solution of K_2PtCl_4 (412 mg, 1.0 mmol, 1.0 eq). The reaction mixture was stirred at room temperature overnight. The obtained yellow solid were collected by filtering, washed with water, ethanol and diethyl ether, and dried in vacuum to give products cis-[$Pt(DMSO)_2Cl_2$] (Yield: 80%).

Preparation of cis-[$PtCl_2$(dppz)].The 5,6-dimethyl-1,10-phenanthroline (0.85 g, 2.8 mmol, 1.1 eq) was dissolved in DMSO (100 mL) and the mixture was added dropwise in DMSO solution of cis-[$Pt(DMSO)_2Cl_2$] (1.05 g, 2.5 mmol, 1.0 eq), The mixture was stirred for 1 h at 50 °C, then filtered. The obtained products cis-[$PtCl_2$(dppz)] were washed with water and a few drops of MeOH, and dried. (Yield: 62%).

Synthesis of 56MESS.(1S, 2S)-cyclohexane-1,2-diamine (114 mg, 1.0 mmol, 1.0 eq) was added to a solution of cis-[PtCl2(dppz)] (473 mg, 1.0 mmol, 1.0 eq) in V(MeOH):V(Water)=1:1 (200 mL). The solution was refluxed for 48 hours. The reaction mixture was concentrated in vacuum and the residue was dissolved in a few drops MeOH.The yellow solid was obtained by recrystalization. (Yield: 42%). The 56MESS was characterized by nuclear magnetic resonance (NMR), high-resolution mass spectrometry (HRMS).

1.3.3. Formulation of NP^{PDT} and NP^{PDT}-56MESS

Briefly, a stirred solution of P1 (20.0 mg) in DMF (1 mL) was dropwise added to deionized water (9 mL). The above liquid was dialyzed in a dialysis bag (MWCO: 3500 Da) for 24 h. NP^{PDT} was obtained. NP^{PDT}-56MESS was obtained in the similar way as NP^{PDT}. A stirred solution of P^{PDT} (20.0 mg) and 56MESS (5.0 mg) in DMF (1 mL) was dropwise added to deionized water (9 mL). The above liquid was dialyzed in a dialysis bag (MWCO: 3500 Da) for 24 h, NP^{PDT}-56MESS was obtained.

1.3.4. Particle size and morphology, and storage stability

The size distribution of NP^{PDT} or NP^{PDT}-56MESS was detected by DLS at the concentration of photosensitive unit (M1) at 80 μg mL^{-1} in aqueous solution, and the morphology of NP^{PDT}-56MESS was observed using TEM. Subsquently, the size distribution of NP^{PDT} or NP^{PDT}-56MESS in PBS was monitored by DLS at the designated time (0 w, 2 w, 4 w).

1.3.5. Detection of ROS generation in aqueous solution

For DPBF assay, the absorbance of DPBF at 415 nm was adjusted to about 1.0 in DCM, the absorbance of NP^{PDT}-56MESS were adjusted to about 0.2. Then, the cuvette was irradiated with 808 nm monochromatic light for various time, and absorption spectra were measured immediately. The absorbance changes of DPBF at 415 nm was used to quantify decomposition rate.

1.3.6. Cell culture

OCM-1 cells and B16-F10 cells were cultured in DMEM (glucose 4.5 g/L) supplemented with 10% fetal bovine serum and 1% P/S at 37 ℃ with 5% CO_2. When the degree of cell fusion reached 80%-90%, the cells were digested with 0.25% trypsin, and then sub-cultured or inoculated in cell plates for subsequent experiments.

1.3.7. Nanoparticles uptake in the cells by CLSM and FCM

A cover slide was placed in the bottom of each well of a 24-well plate. Cells (1×10^5) in 1 mL medium were added to each well and incubated at 37 °C for 12 h. Then, the cells were incubated with NP^{PDT}-56MESS @Cy5.5 (1.25 μM Pt) diluted in cell media for various time intervals (0 h, 1 h, 4 h, 7 h). Next, the cells were washed with phosphate-buffered saline (PBS) and further incubated with the nucleus specific stain DAPI (ab285390, Abcam) and the cytoskeleton specific stain Alexa-488 (1:500, Beyotime) for 0.5 h. Subsequently, the cellular uptake was assessed by CLSM (DAPI, λex = 405 nm, λem = 460 nm, Cy5.5, λex = 673 nm, λem = 692 nm). The Origin software were used to analysis and draw the pictures.

To perform flow cytometry, cells were seeded into 12-well plate at 2×10^5 cells/well and incubated at 37 °C for 12 h. The NP^{PDT}-56MESS@Cy5.5 (1.25 μM Pt) were added to each well in different time points (1 h, 4 h, 7 h), and the wells without any treatment were performed as negative control. Afterwards, the cells were harvested and quantified using FCM.

1.3.8. Intracellular ROS generation

A cover slide was placed in the bottom of each well of a 24-well plate. Cells (1×10^5) in 1 mL complete media were added to each well and incubated at 37 °C for 12 h. Afterward, the cells were treated with NP^{PDT} and NP^{PDT}-56MESS at the photosensitive unit at 20 μg/mL for 7 h respectively. Subsequently, the culture medium of the cells was replaced with a serum-free medium and then incubated with ROS indicator DCFH-DA (10 μM) for 20 mins. Then the cells of laser groups were irradiated with a NIR light at 808 nm after washing with PBS. Afterward, the cover slide of each well was placed on the microslide, and the cell nuclei were stained with DAPI. Subsequently, images were collected with CLSM.

Furthermore, the intracellular ROS level was further detective and quantify by FCM. First, cells were seeded in 12-well plate at a density of 2×10^5 per well and incubated at 37 °C for 12 h. Afterward, the cells were treated with the same conditions as the above CLSM analysis. Finally, the cells were harvested to examine the intracellular DCF by FCM.

1.3.9. *In vitro* cytotoxicity assays

Cells (5×10^3 cells/well) were seeded into 96-well plate and treated with various agents for the cytotoxicity study. After 12 h incubation, the cells treated with NP^{PDT} + L or NP^{PDT}-56MESS + L were irradiated with NIR light of 808 nm at intensity of 1.0 W cm^{-2} for 3 min. After incubation for another 12 h, the viability was analyzed by MTT colorimetric assay.

1.3.10. mtDNA Stress Assay

Cover slides were placed in the bottom of each well of a 24-well plate, and OCM-1 cells (1×10^5) in 1 mL media were added to each well and incubated at 37 °C for 12 h. Cells were then treated with PBS, 56MESS, NP^{PDT} , NP^{PDT}-56MESS, NP^{PDT} + L or NP^{PDT}-56MESS + L (Pt at 1.25 μM, photosensitive unit 10 μM) for 12 h. Subsequently, the cells were washed and permeabilized. Next, Anti-TOMM20 antibody (MA5-34964, Thermo Scientific™) was incubated to stain mitochondria for 2 h at 37°C, and then with goat anti-rabbit secondary antibody for 1 h. dsDNA Marker (HYB331-01, Santa Cruz Biotechnology) was incubated in the same way.

1.3.11 qPCR Quantification of Cytosolic mtDNA

OCM-1 cells were cultured overnight in cell culture dishes and then treated with PBS, 56MESS, NP^{PDT} , NP^{PDT}-56MESS, NP^{PDT} + L or NP^{PDT}-56MESS + L (Pt at 1.25 μM, photosensitive unit 10 μM) for 12 h. After being washed with PBS, the cells were collected by centrifugation. Cytosolic mtDNA was removed using a Cell Mitochondria Isolation Kit (89874, Thermo Scientific™), and the DNA was then extracted with a TIANamp Genomic DNA kit (K0721, Thermo Scientific™) following the manufacturer's protocol. The mtDNA DN-1 primers [3] (Table S3) and TransStart Top Green qPCR SuperMix (AQ131-03 ,TransStart®) were employed for qPCR. The amount of cytosolic mtDNA was normalized to β -actin. [4]

1.3.12. Immunofluorescence staining

Cover slides were placed in the bottom of each well of a 24-well plate, and OCM-1 cells (1×10^5) in 1 mL media were added to each well and incubated at 37 °C for 12 h. Cells were then treated with PBS, 56MESS, NP^{PDT}-56MESS, NP^{PDT} + L or NP^{PDT}-56MESS + L (Pt at 1.25 μM, photosensitive unit 10 μM) for 12 h or 24 h, washed with PBS, and fixed in 4% paraformaldehyde solution. Thereafter, cells were blocked with 1% BSA (Beyotime), penetrated by 0.1% Triton (Beyotime) and then incubated with γ -H2AX and p-STING, primary antibody at 4℃ for 12 h, respectively. After washing with PBS for 3 times, cells were incubated with Alexa Fluor 555-conjugated antibody (ab302576, Abcam) for 3 h. Nuclei were counterstained with DAPI and then the stained sections were imaged with CLSM.

1.3.13 Dendritic cells activation *in vitro*

To evaluate dendritic cells (DCs) activation *in vitro*, bone marrow-derived dendritic cells (BMDCs) were obtained from the bone marrow of mice and cultuted in RPMI 1640 medium

supplement with 10% FBS, granulocyte-macrophage colony-stimulating factor (GM-CSF) (20 ng/mL, Beyotime), and interleukin-4 (IL-4) (10 ng/mL, Beyotime) at 37 °C with 5% (v/v) CO_2. After 5 days culturing, pretreated B16-F10 cells were co-incubated with BMDCs for 24 h. After that, the BMDCs were collected and stained with anti-CD11c, anti-CD80 and anti-CD86 antibodies. Finally, the activation of DCs was examined by using FCM measurement.

1.3.14 ELISA assay

OCM-1 cells were treated with PBS, 56MESS, NP^{PDT}-56MESS, NP^{PDT} + L or NP^{PDT}-56MESS + L (Pt at 1.25 μM, photosensitive unit 10 μM) diluted in cell media for 24 h, then the drug pretreated tumor cells supernatant were obtained after centrifugation at a speed of 12000 rpm/min for 5 min. To test the cytokines levels produced by cells stimulated with different treatments, the above supernatant was prepared for cytokines analysis using Mouse IL-6 uncoated ELISA kits (88-7064-88, ThermoFisher) and Mouse IFN-β ELISA Kit (EK2236-96, Multi Sciences), which were carried out as manufacturer's instructions.

The peripheral blood was collected from mice treated with different drugs for ELISA tests through centrifuging at a speed of 12000 rpm/min for 5 min. The above peripheral blood was prepared for cytokines analysis using Mouse IL-6 uncoated ELISA kits , Mouse IFN-β ELISA Kit and Mouse IFN-γ ELISA Kit (EK280/3-96, Multi Sciences). The test procedures strictly followed the manufacturer's instructions.

1.3.15 Western blotting

OCM-1 cells were treated with PBS, 56MESS, NP^{PDT}-56MESS, NP^{PDT} + L or NP^{PDT}-56MESS + L (Pt at 1.25 μM, photosensitive unit 10 μM) diluted in cell media for 24 h . The above OCM-1 cells were added with RIPA lysis buffer (P0013B, Beyotime) with protease and phosphatase inhibitors (P1097, Beyotime). The proteins of OCM-1 cells were extracted *via* centrifugation at a speed of 12000 rpm for 15 min. The concentration of protein was measured by the BCA protein assay kit (P0011, Beyotime). Proteins in equivalent amounts were then separated on a 10% sodium dodecyl sulfate-polyacrylamide gel electrophoresis (SDS-PAGE) and transferred to the PVDF membrane by a gel-electrophoretic apparatus (Bio-Rad mini, USA), followed by blocking in TBS-T solution containing 5% skim milk for 1 h. Subsequently, the membrane was incubated with primary antibody against STING (#13647, CST), p-STING (#50907, CST), TBK1 (#38066, CST), p-TBK1 (#5483, CST), IRF3 (#4302, CST), p-IRF3 (#29047, CST) overnight on a shaker at 4 ℃. Subsequently, the PVDF films were washed 3 times for 30 min and incubated with HRP (A0208, Beyotime) conjugated antibodies for 2 h at room temperature. The Western blot images were obtained by Gel imaging system (Tanon 4800, China) with 200 μL of ECL chemiluminescent reagent (KF001, Affinity) added on the top of the membrane. α-tubulin (#2144, CST) were employed as protein loading control.

1.3.16. Animal welfare and protocols

Healthy KM mice, BALB/c nude mice, and C57BL/6 mice were purchased from SPF Biotechnology (Beijing, China) and raised in SPF animal rooms. All animal experiments reported herein were performed under guidelines evaluated and approved by Peking University Institutional Animal Care and Use Committee (LA2021316).

1.3.17. *In vivo* biosafety evaluation

Healthy KM mice were randomly grouped (n = 3 mice per group). Each mouse was injected with a single dose of PBS, NP^{PDT}, 56MESS and NP^{PDT}-56MESS (Pt at 0.4 /kg body weight, photosensitive unit at 10 mg/kg body weight). The mice were monitored and weighed on every three days. Then the mice were sacrificed at 14 days after administration, and the blood samples of the mice were collected for hematological and serum biochemical analyses. Meanwhile, the main organs were also dissected from the mice for further analysis after being sacrificed. All the tissues were paraffin embedded and tissue sections were prepared. Hematoxylin and eosin (H&E) staining was performed on the tissue sessions to observe pathological features, and images were captured using a fluorescence microscope (IX83, Olympus).

1.3.18. *In vivo* biodistribution imaging.

The NP^{PDT} -56MESS@cy7.5 (photosensitive unit at 10 mg/kg body weight) was intravenously injected into the tumor-bearing mice. Afterward, the mice were anesthetized for *in vivo* biodistribution imaging at different interval time after injection. Biodistribution images were collected by IVIS (Spectrum CT, PerkinElmer, E_x/E_m=745 nm/840 nm) at various time points. The mice were sacrificed after 48 h of nanoparticles injection. *Ex vivo* imaging of organs including heart, liver, spleen, lung, kidney and tumor were collected and quantitative analyses using IVIS Spectrum imaging system.

1.3.19. Establishment of OCM-1 Uveal Melanoma orthotopic model

OCM-1 cells (5×10^5) were dispersed in PBS buffer and implanted into right tibia of BALB/c mice to establish an orthotopic tumor-bearing model. One week after the tumor was implanted, the tumor was grown to about 70 mm^3.

1.3.20. Histological and immunohistochemical analyses

After two consecutive treatments, some mice were humanely sacrificed, followed by the excision of tumors for histological observation by standard H&E staining and immunofluorescence (IF) staining. For H&E staining, the excised tumor were fixed in 4% paraformaldehyde solution, embedded in paraffin, sectioned and stained with hematoxylin and eosin. The sections were then observed under a fluorescence microscope (IX83, Olympus). TUNEL staining was also used to evaluate apoptosis of tumor tissues, the procedures were consistent with the manufacturer’s protocol, and finally examined by CLSM. For detecting the expression of p-STING, and infiltration of $CD8^+$ T cells in tumor tissues, frozen tumor sections were fixed, and blocked with 1% BSA. Then the sections were incubated with primary antibodies against p-STING, and CD8 (MA1-84018, Invitrogen) overnight at 4 ℃, followed by processing with corresponding second antibodies. Nuclei were counterstained with DAPI (ThermoFishere Scientific) and then the stained sections were imaged by CLSM.

1.3.21. Flow cytometry analysis of the animal tissue

Female C57BL/6 mice (6-8 weeks old) were implanted with B16-F10 cells ($1\times10^{5)}$. Once the tumor volume reached approximately 70 mm^3, the mice were assigned at random (n = 3) and received the same treatment as previously described. Mice were sacrificed at day 7. The obtained tumors, spleens and draining lymph nodes were used to prepare single cell suspensions. The single-cell suspensions were further incubated with various antibodies against the immune cells. FCM analysis was carried out on the FCM machine, and the results were analyzed with the FlowJo_V10 software.

To detect the impacts on DCs maturation in mice, The single cell suspension from tumor tissues and tumor-draining lymph nodes (TDLNs) were respectively stained with primary antibodies of anti-CD11c-PE, anti-CD80-FITC and anti-CD86-APC for FCM analysis. The matured DCs were denoted as $CD11c^+CD80^+CD86^+$ cells. To detect the infiltration of $CD8^+$ T cells, the single cell suspension from tumor tissues or spleens were incubated with anti-PE-CD3, anti-CD8-FITC and anti-CD4-APC for FCM analysis. The $CD8^+$ T cells were marked as $CD3^+CD4^-CD8^+$ T cells. TCM ($CD44^+CD62L^+$) in spleens and tumor-draining lymph nodes, NKs ($CD69^+$ $NK1.1^+$) and Tregs ($CD4^+Foxp3^+$) in tumor tissues were also evaluated.

1.3.22. Statistical analysis

Data were presented as mean ± SD. Statistical significances for comparison between two groups, student's t-test was used and for comparison between multiple groups, one-way ANOVA was used. Differences were considered statistically significant at a level of $*p < 0.05$; $**p < 0.01$; $***p < 0.001$; $****p < 0.0001$**.**

1.4. Supplement References

[1] W. Dengshuai, Y. Yingjie, H. Yun, J. Yuming, Z. Yao, N. Zongxiu, W. Fuyi, M. Wen, Y. Zhiqiang, H. Yuanyu, Z. Xiao-Dong, L. Zhao-Qian, Z. Xingcai, X. Haihua, *ACS Nano* **2021**, https://doi.org/10.1021/acsnano.1c00076.
[2] T. Wu, J. Liu, M. Liu, S. Liu, S. Zhao, R. Tian, D. Wei, Y. Liu, Y. Zhao, H. Xiao, B. Ding, *Angew Chem Int Ed Engl* **2019**, *58* (40), 14224, https://doi.org/10.1002/anie.201909345.
[3] C. S. Lin, S. C. Chang, L. S. Wang, T. Y. Chou, W. H. Hsu, Y. C. Wu, Y. H. Wei, *J Thorac Cardiovasc Surg* **2010**, *139* (1), 189, https://doi.org/10.1016/j.jtcvs.2009.04.007.
[4] X. Zhao, Y. Wang, W. Jiang, Q. Wang, J. Li, Z. Wen, A. Li, K. Zhang, Z. Zhang, J. Shi, J. Liu, *Adv Mater* **2022**, *34* (37), e2204585, https://doi.org/10.1002/adma.202204585.

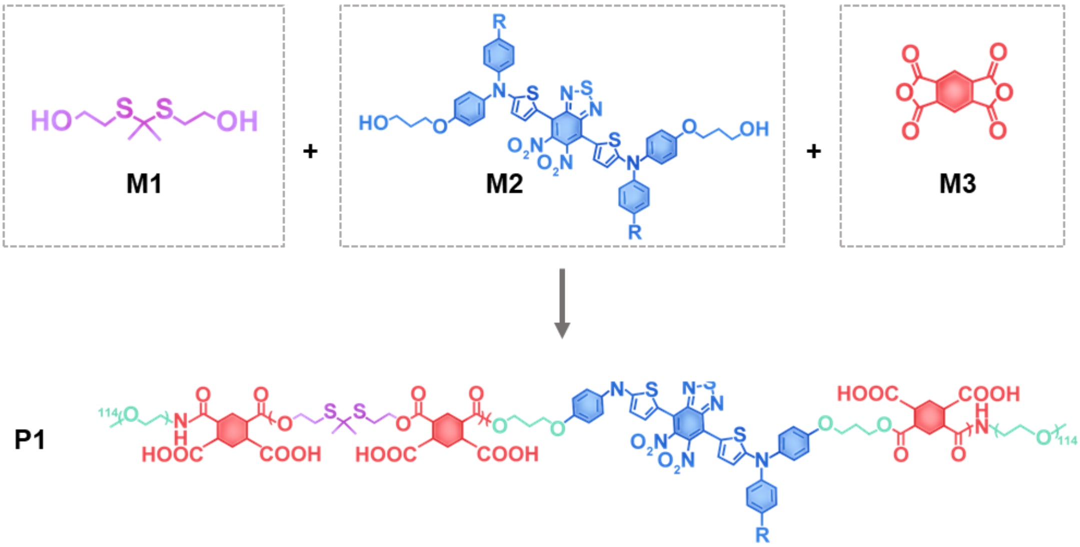

Scheme S1. Synthesis route of P1. P1 was synthesized as previously described [1].

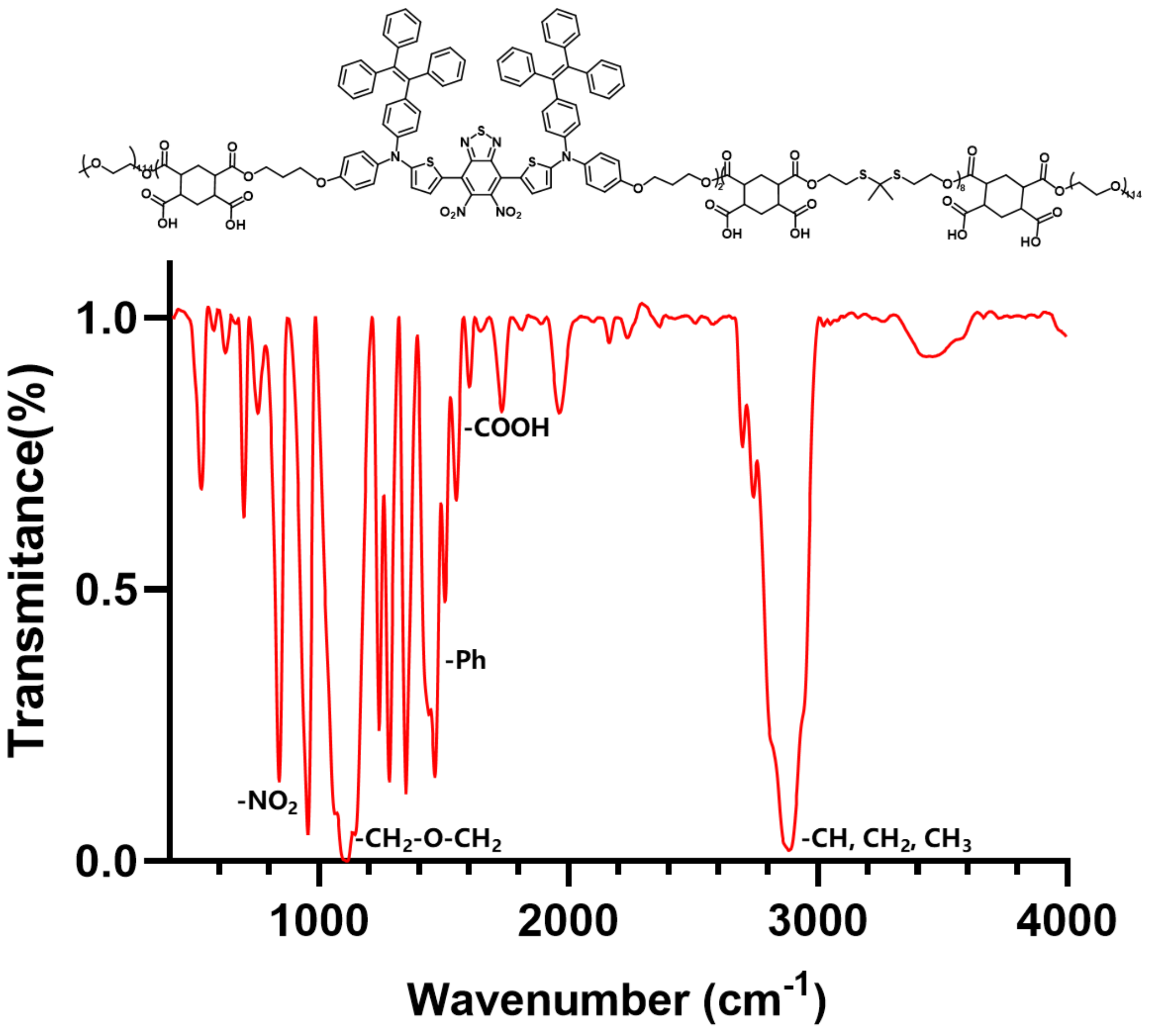

Figure S1. Infrared radiation (IR) of P1.

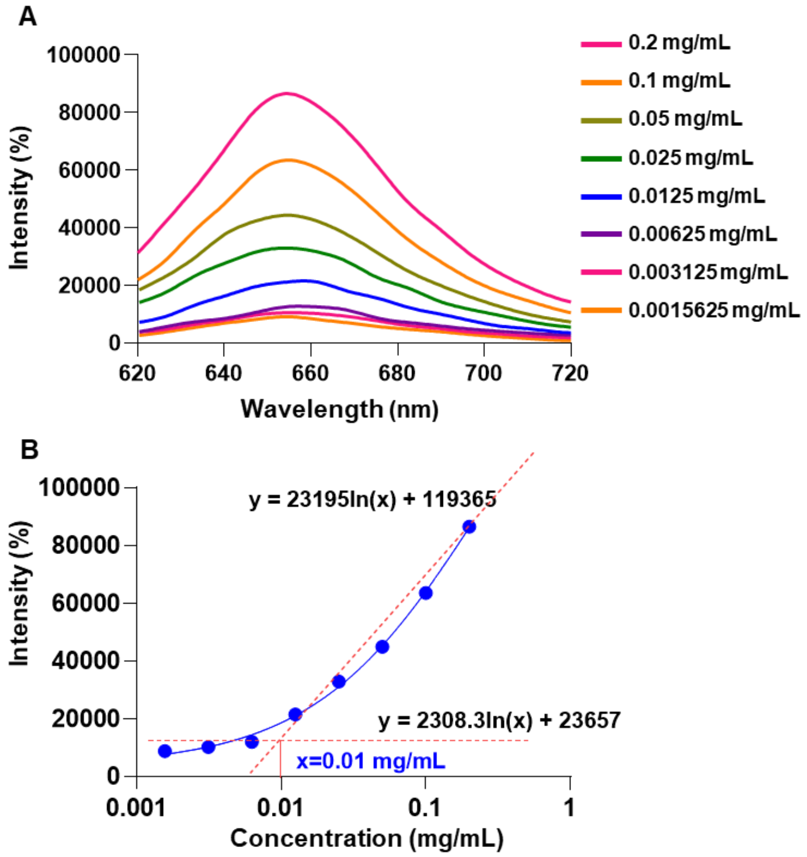

Figure S2. Determination of the critical micelle concentration (CMC) of P1. (A) The fluorescence spectrum of P1 at different concentrations encapsulated with predetermined amount of Nile red; (B) The peak fluorescence at 652 nm versus polymer concentrations was plotted for CMC measurement (CMC = 0.01 mg/ml).

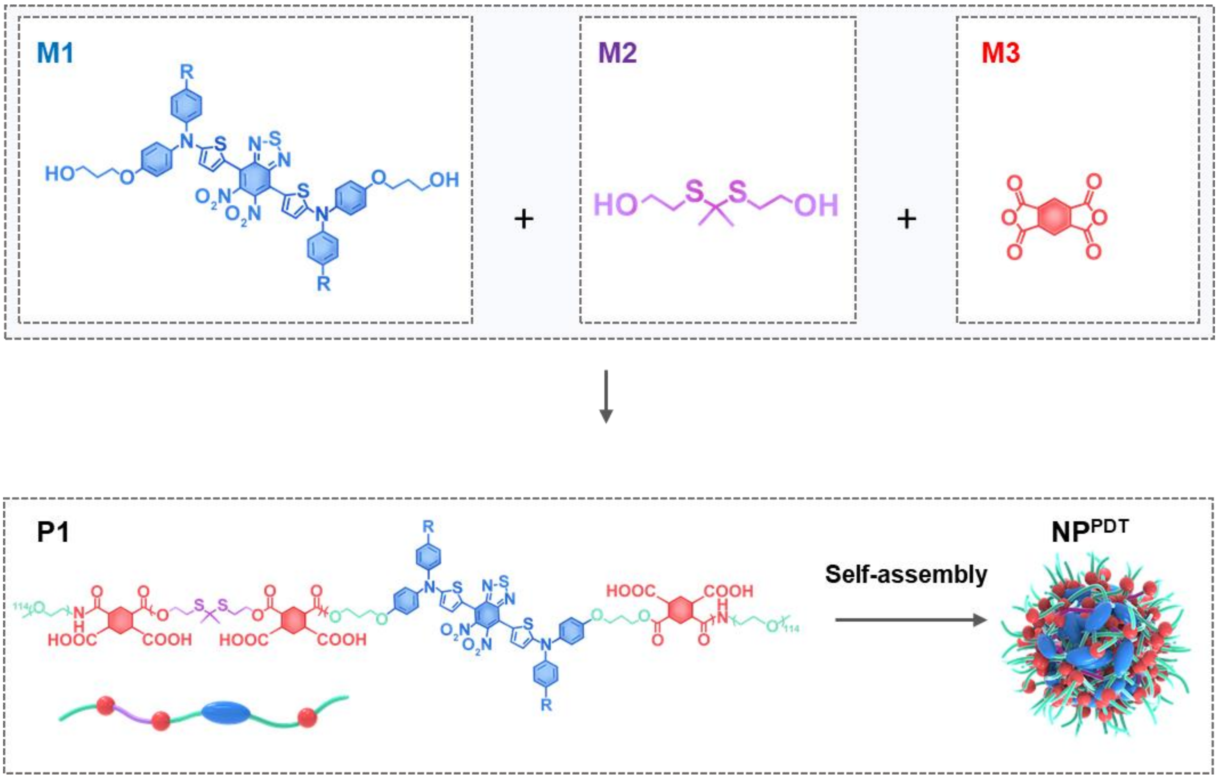

Scheme S2. Synthesis route of NP^{PDT}.

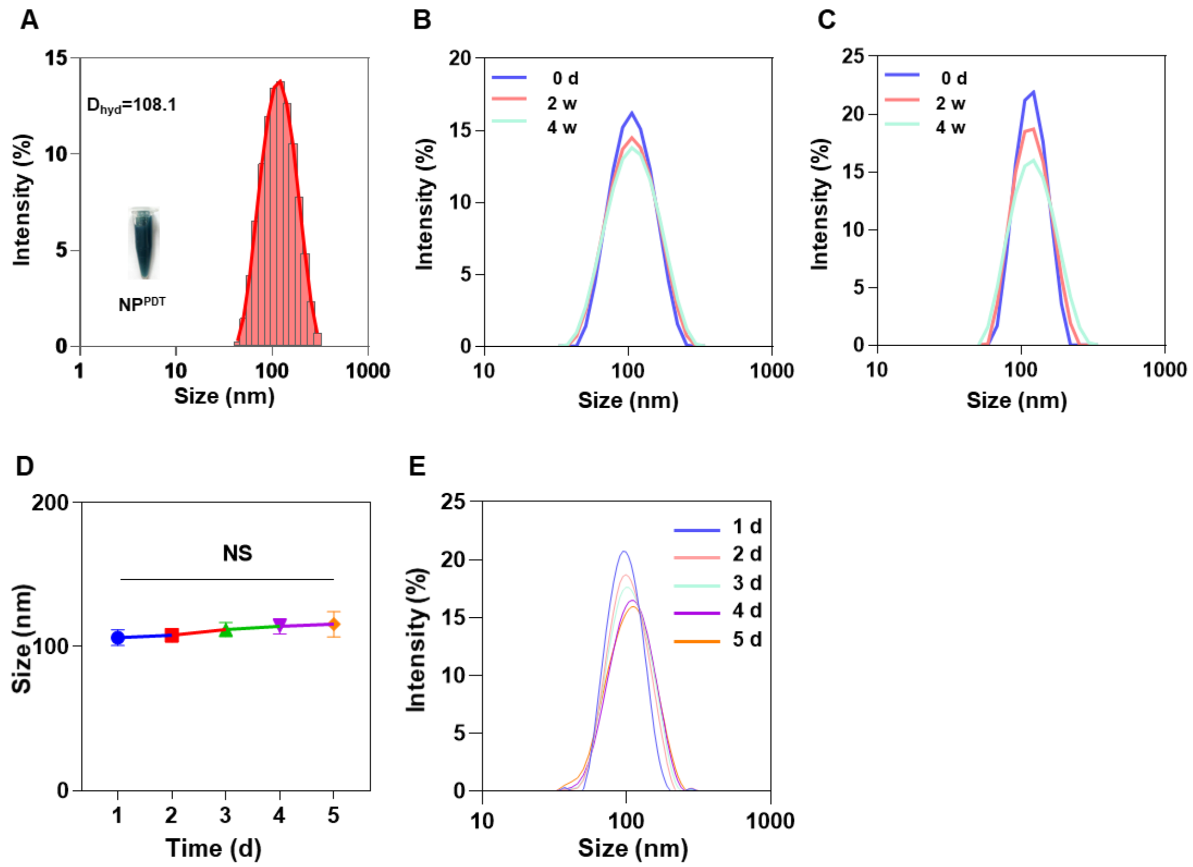

Figure S3. characterization of NP^{PDT} and NP^{PDT}-56MESS. (A) The diameter of NP^{PDT} by DLS. The diameter of NP^{PDT} (B) and NP^{PDT}-56MESS (C) throughout four weeks of storage in PBS by DLS. the particle size (D) and size distribution (E) of NP^{PDT}-56MESS throughout five days of storage in serum by DLS. (n=3). Data are presented as mean ± SD. Statistical significances between every two groups were calculated via one-way ANOVA.

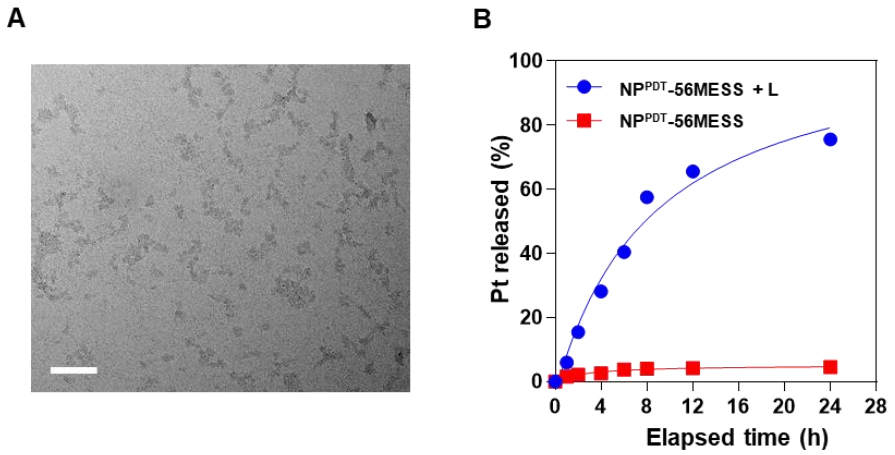

Figure S4. NP^{PDT}-56MESS disintegrated upon laser irradiation. (A) Representative image of NP^{PDT}-56MESS after NIR laser irradiation (808 nm 1W x cm^{-2} x 3 min) for 180 s by TEM. Scale bar: 100 nm. (B) Representative 56MESS release profiles of NP^{PDT}-56MRSS with or without NIR laser.

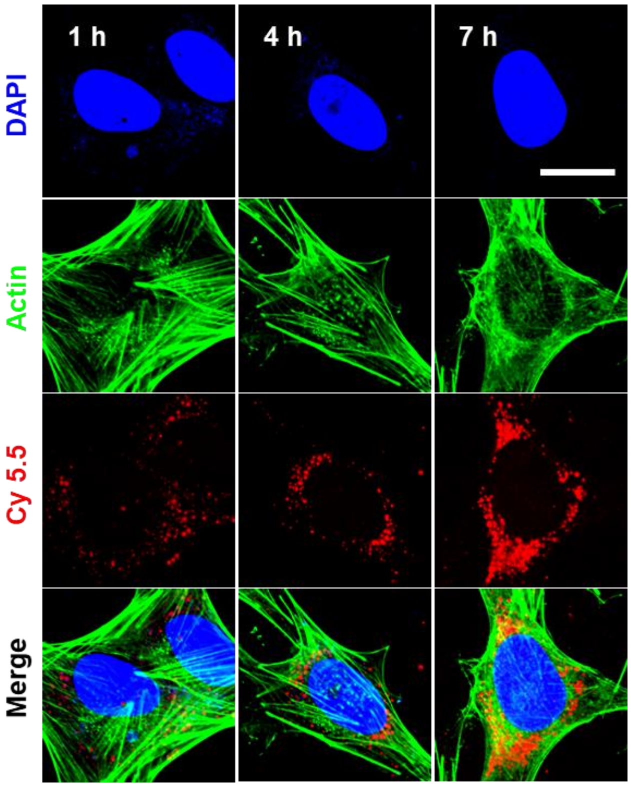

Figure S5. Intracellular uptake of Cy5.5-labeled NP^{PDT}-56MESS at 1 h, 4 h, and 7 h by CLSM. (Blue, DAPI; Green, Actin, Red, Cy 5.5). Scale bar: 20 μm.

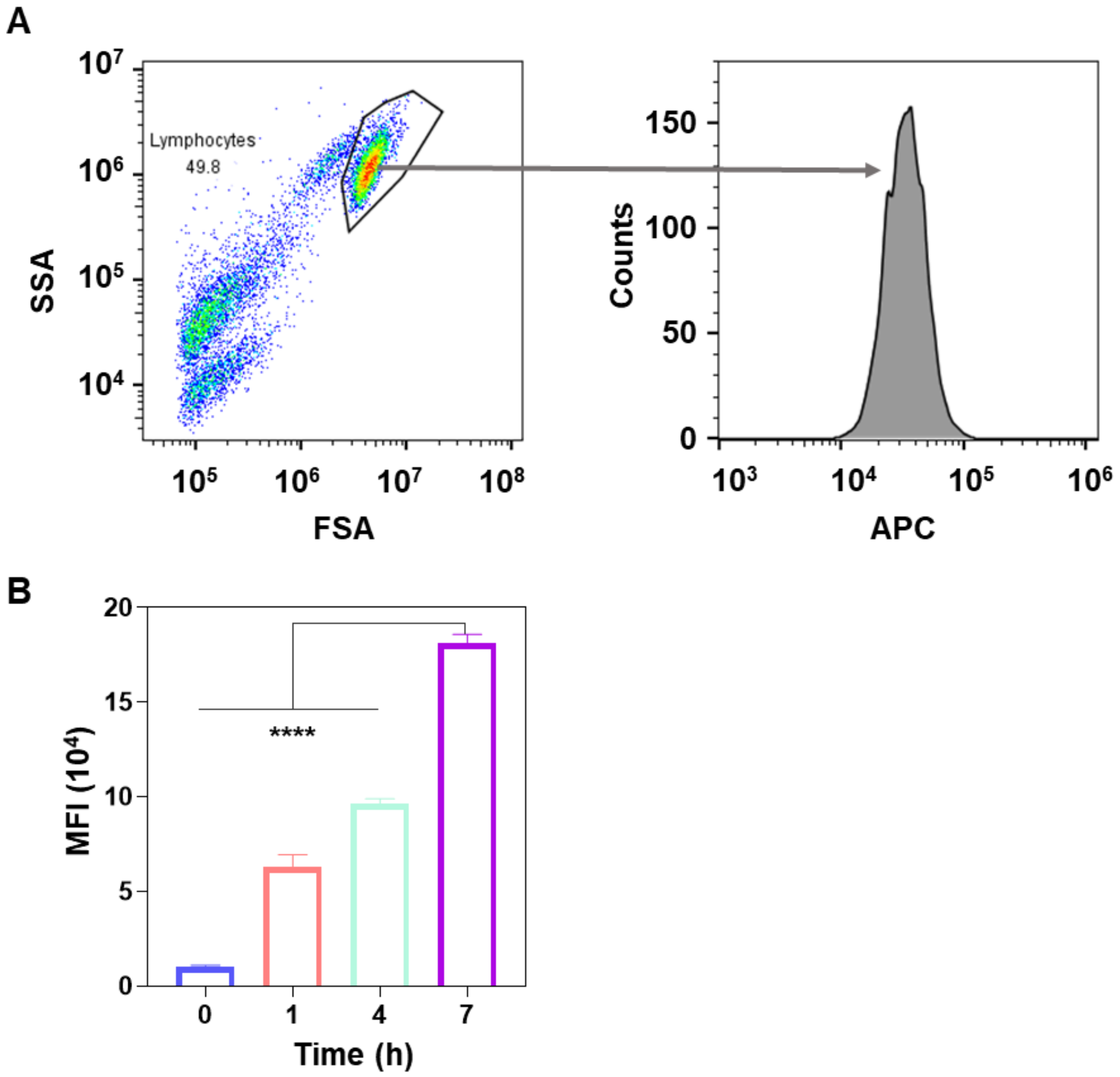

Figure S6. Intracellular uptake of Cy5.5-labeled NP^{PDT}-56MESS at 1 h, 4 h, and 7 h by FCM. (A) The gating strategy. (B) Quantification of intracellular uptake of Cy5.5 labeled NP^{PDT}-56MESS. (n=3). Data are presented as mean ± SD. Statistical significances between every two groups were calculated via one-way ANOVA.

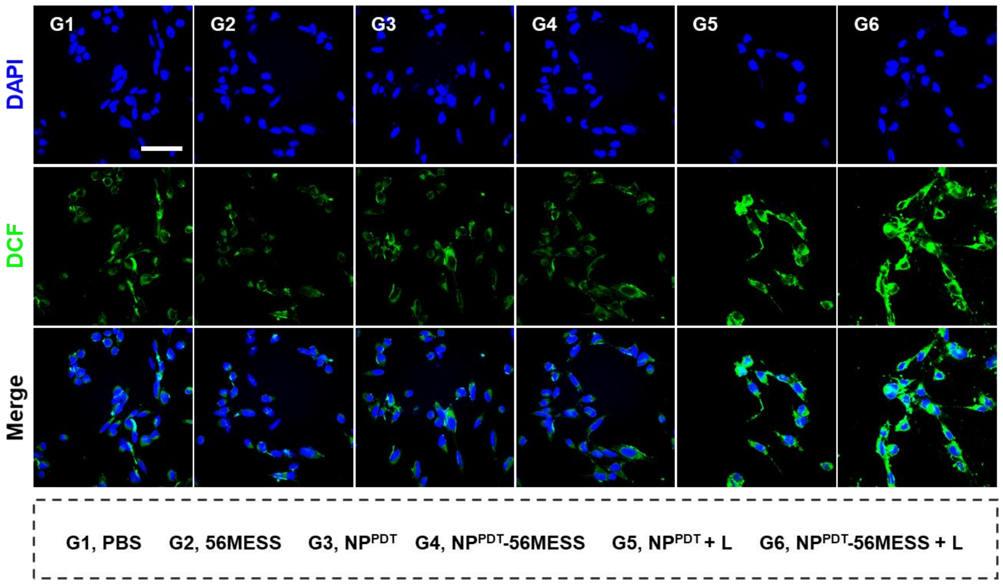

Figure S7. The ROS generation in OCM-1 cells treated with various durgs in the dark or upon light irradiation (808 nm 1W x cm^2 x 3 min) by CLSM. (Blue, DAPI; Green, DCF) Scale bar: 60 μm.

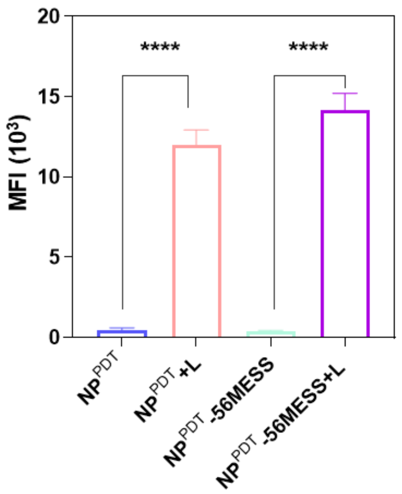

Figure S8. The ROS generation in OCM-1 cells treated with various durgs in the dark or upon light irradiation (808 nm 1W x cm2 x 3 min) by FCM. (n=3). Data are presented as mean ± SD. Statistical significances between every two groups were calculated via one-way ANOVA.

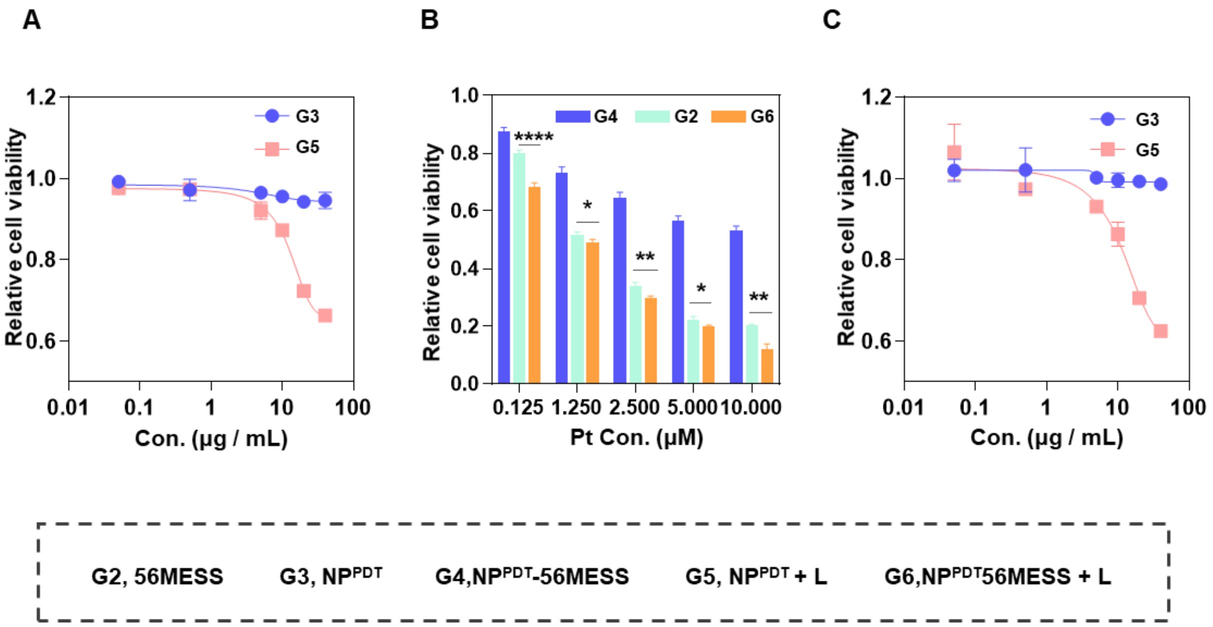

Figure S9. Relative cell viabilities of cells with various treatments. (A) Relative cell viabilities of OCM-1 cells treated with NP^{PDT} or NP^{PDT} + L (808 nm 1W x cm^{-2} x 3 min) *via* an MTT assay at 24 h. The maximum concentration of photosensitive units in NP^{PDT} was 40 ug / mL. (B), (C) Relative cell viabilities of B16-F10 cells with various drugs exposure to irradiation (808 nm 1W x cm^{-2} x 3 min) *via* an MTT assay at 24 h. The maximum concentration of photosensitive units in NP^{PDT} was 40 ug / mL. (n=3). Data are presented as mean ± SD. Statistical significances between every two groups were calculated via one-way ANOVA. *$p < 0.05$, **$p < 0.01$, ****$p < 0.0001$.

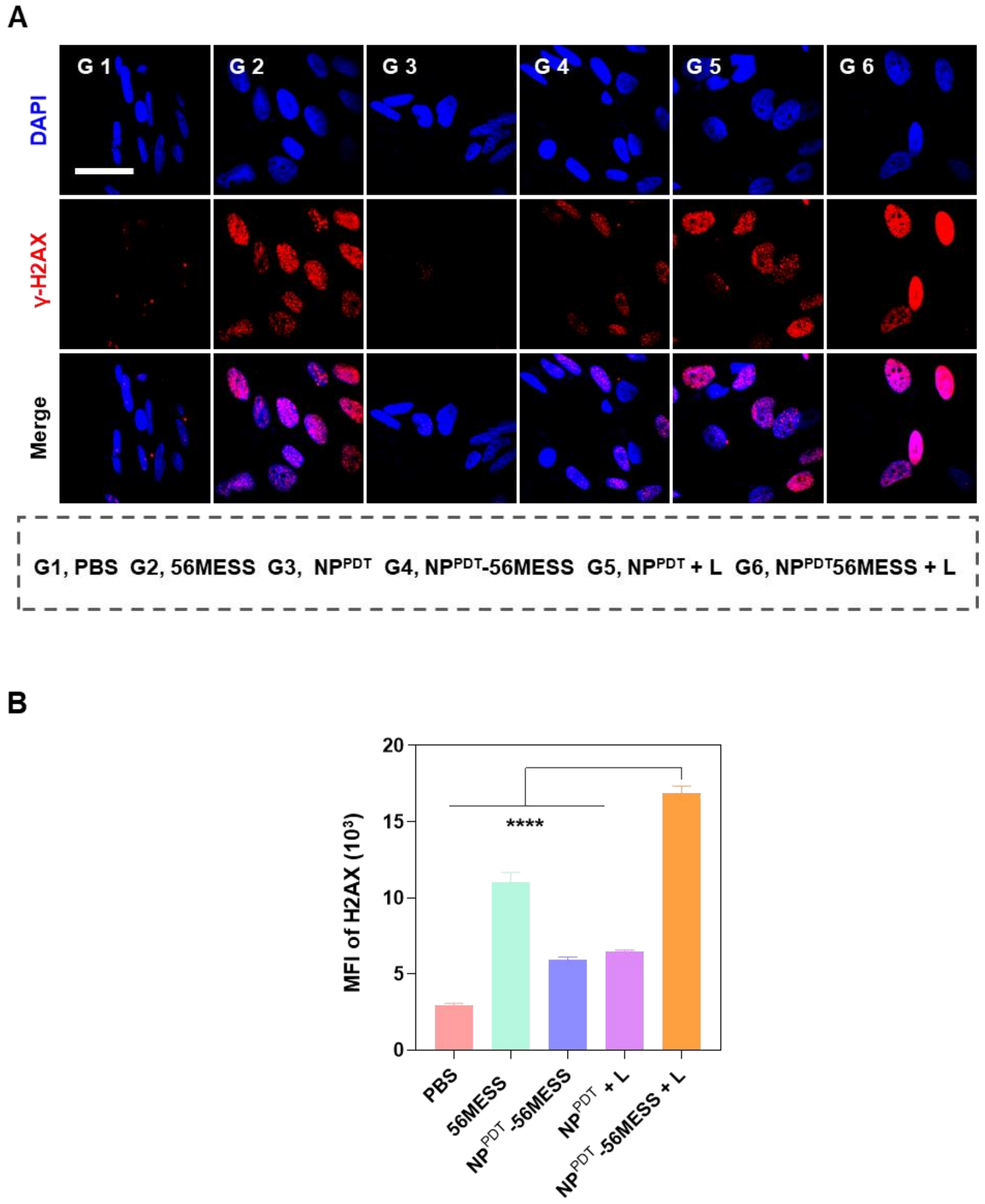

Figure S10. DNA damage occured in OCM-1 cells after various treatments. (A) Representative CLSM images of γ-H2AX in cells treated with various drugs for 12 h (Blue, DAPI; Red, γ-H2AX). Scale bar: 20 μm . (B) Quantification of γ-H2AX in cells treated with various drugs for 12 h. (n=3). Data are presented as mean ± SD. Statistical significances between every two groups were calculated via one-way ANOVA. ****$p < 0.0001$.

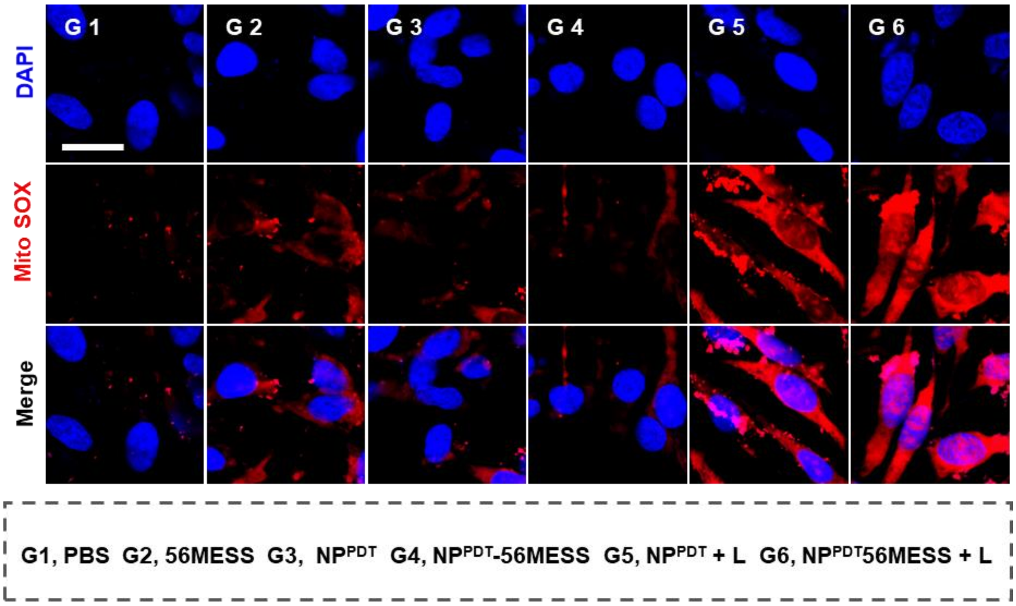

Figure S11. Representative CLSM images of mitochondrial ROS in cells treated with various treatments for 12 h. (Blue, DAPI; Red, MitoSOX ;). Scale bar: 20 μm.

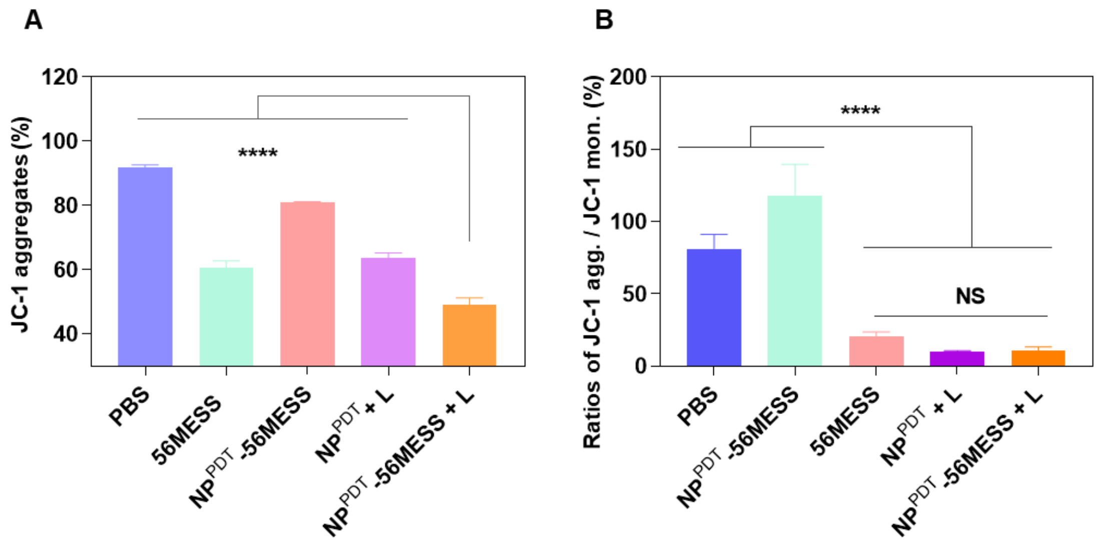

Figure S12. Quantification of mitochondrial membrane potential (MMP) changes in OCM-1 cells. Relative JC-1 aggregates (A) and ratios of JC-1 aggregates ./ JC-1 monomer (B) in cells after different treatments for 12 h by FCM. n=3. Data are presented as mean ± SD. Statistical significances between every two groups were calculated via one-way ANOVA. ****$p < 0.0001$.

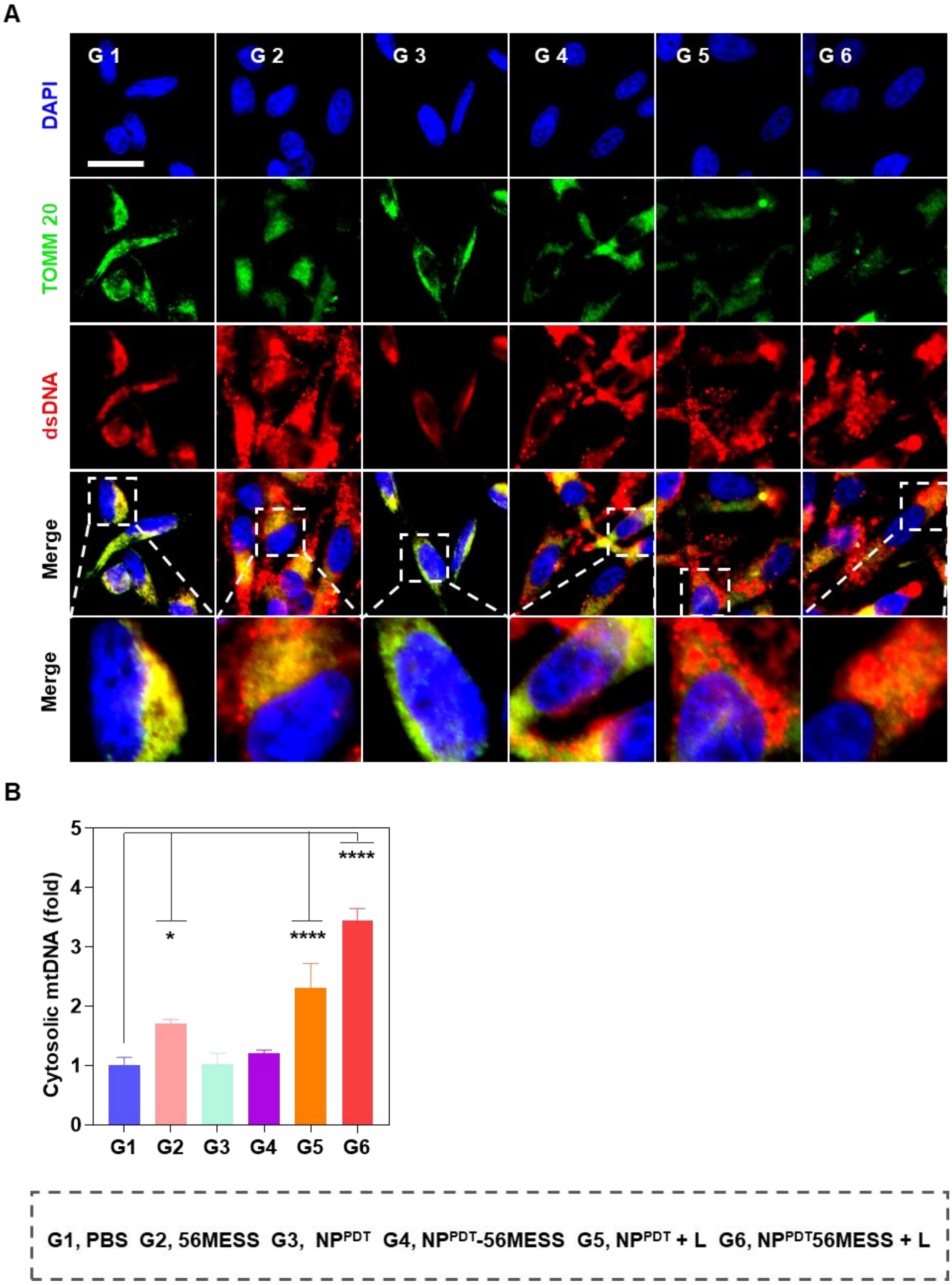

Figure S13. Accumulation of dsDNA in the cytoplasm of cells after various treatments. (A) Representative CLSM images of dsDNA in cells treated with various treatments for 12 h. (Blue, DAPI; Green, mitochondria; Red, dsDNA;). Scale bar: 20 μm. (B) Quantification of Cytosolic mRNA level by qPCR. (n = 3). Data are presented as mean ± SD. Statistical significances between every two groups were calculated via one-way ANOVA. $**p < 0.01$, $****p < 0.0001$.

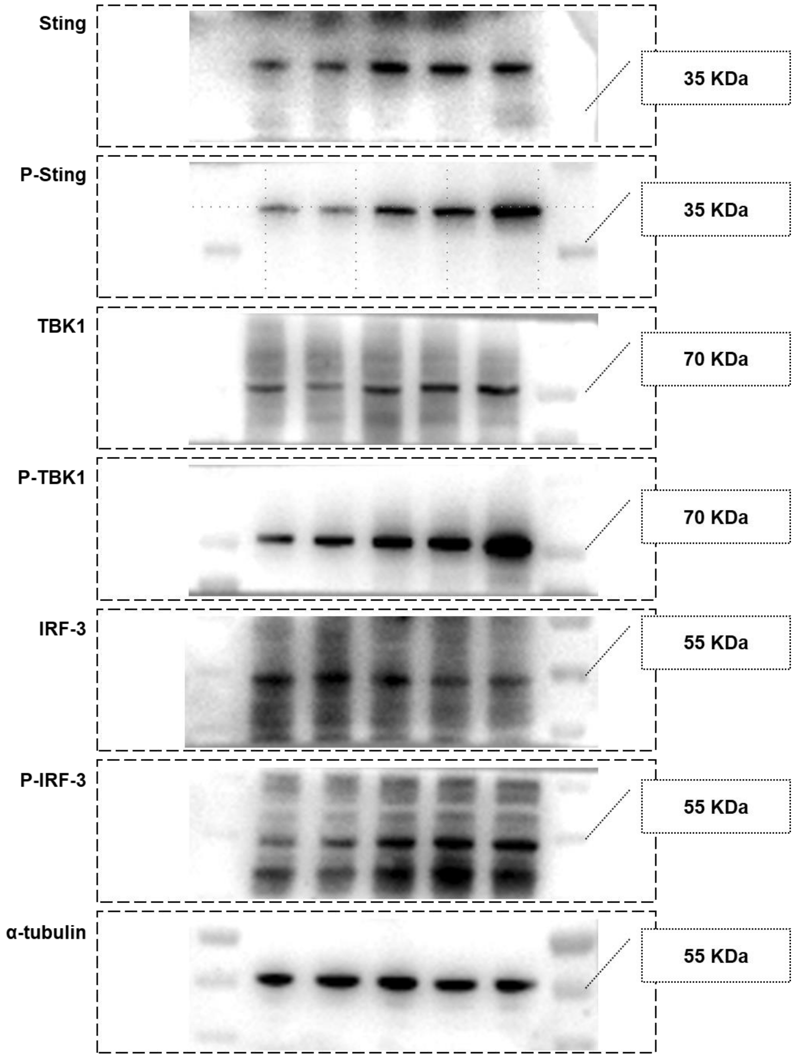

Figure S14. Original western blot images of STING-related proteins after different treatments for 24 h in OCM-1 cells.

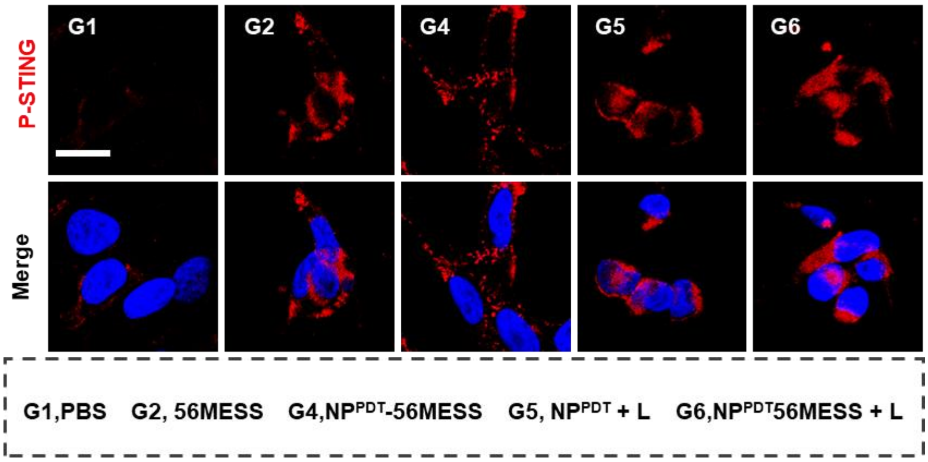

Figure S15. Representative CLSM images of p-STING protein in OCM-1 cells after various treatments for 24 h. (Blue, DAPI; Red, p-STING;). Scale bar: 20 μm.

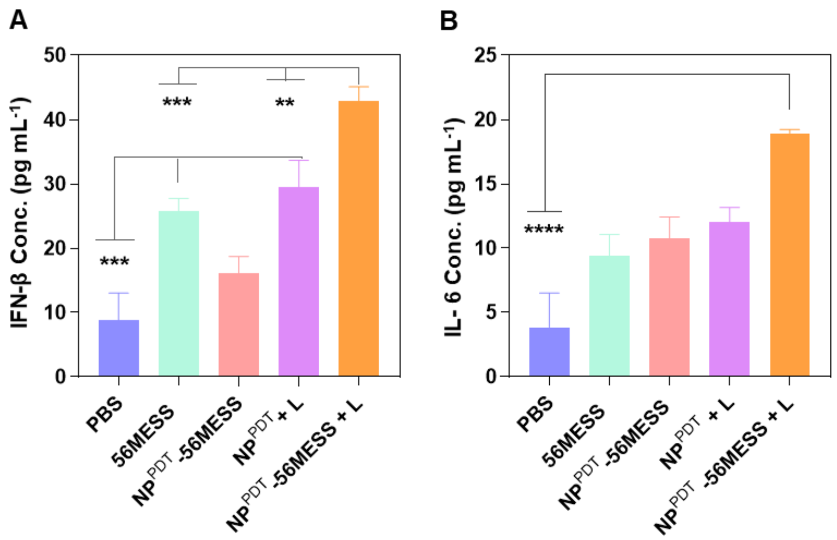

Figure S16. IFN- β (A) and IL-6 (B) levels in OCM-1 cells supernatant after various treatments for 24 h by ELISA. (n=3). Data are presented as mean ± SD. Statistical significances between every two groups were calculated via one-way ANOVA. $^{**}p < 0.01$, $^{***}p < 0.001$, $^{****}p < 0.0001$.

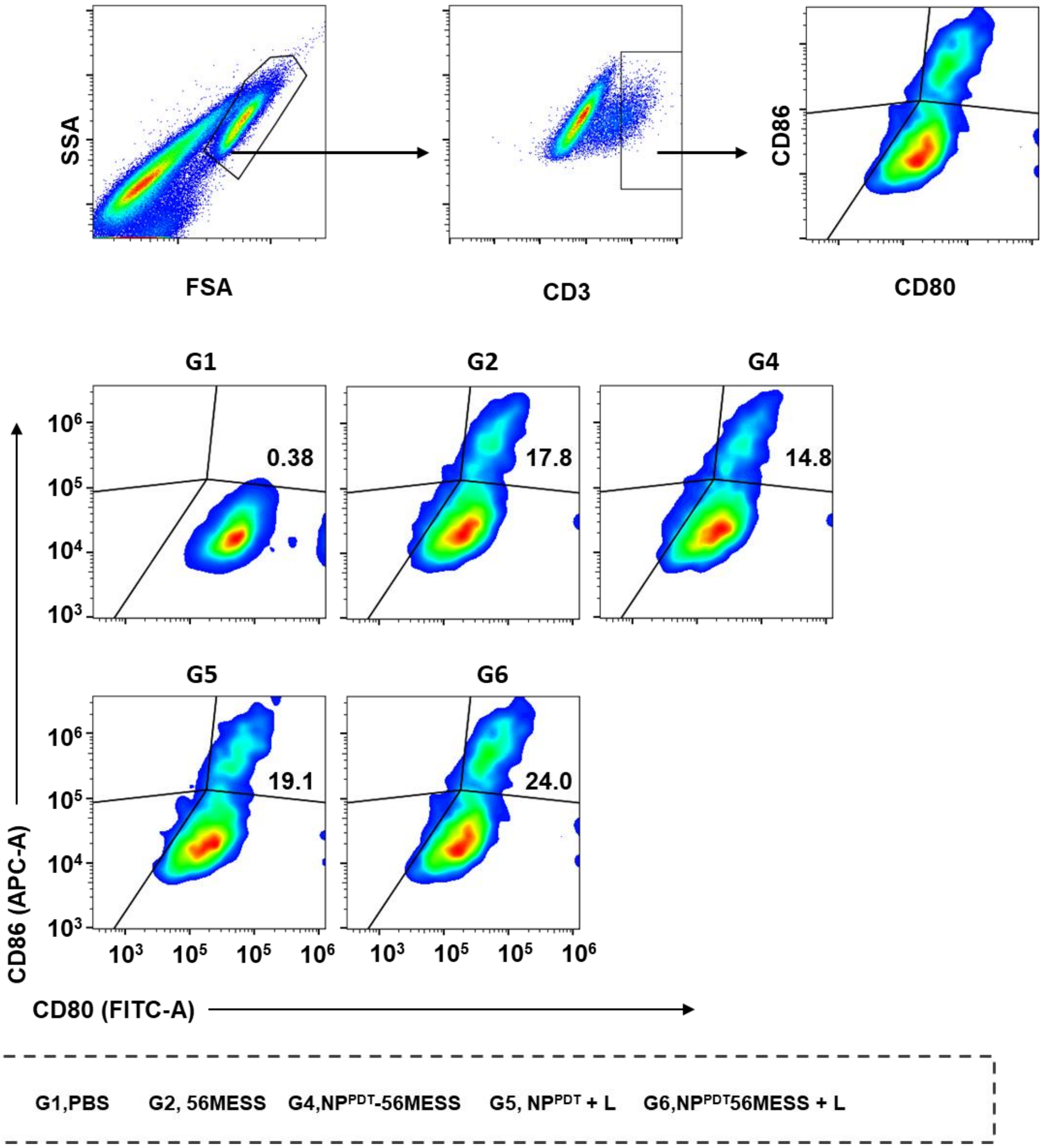

Figure S17. The gating strategy (A) and typical flow cytometer analysis profiles (B) of CD11C^{+}CD80^{+} CD86^{+} DCs cells when co-cultured with B16-F10 cells treated with various drugs for 24 h.

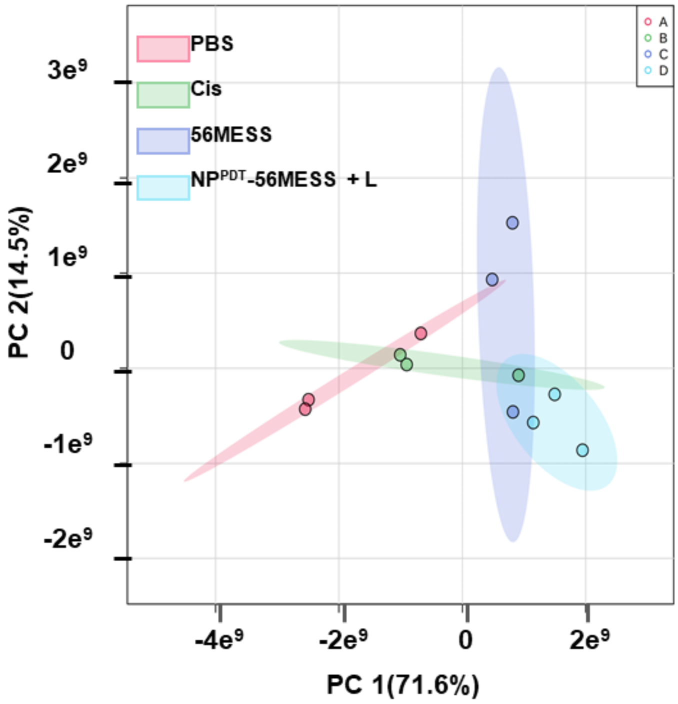

Figure S18. Principal component analysis of all detectable metabolites in OCM-1 cells treated with PBS, Cis, 56MESS, and NP^{PDT}-56MRSS + L . (n=3).

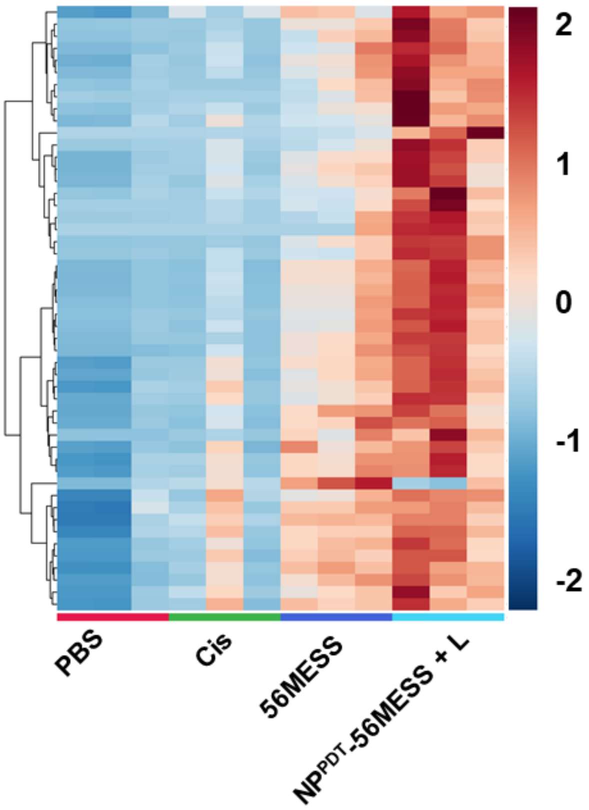

Figure S19. A heat map based on the different metabolites between the OCM-1 cells treated with various agents. (n=3).

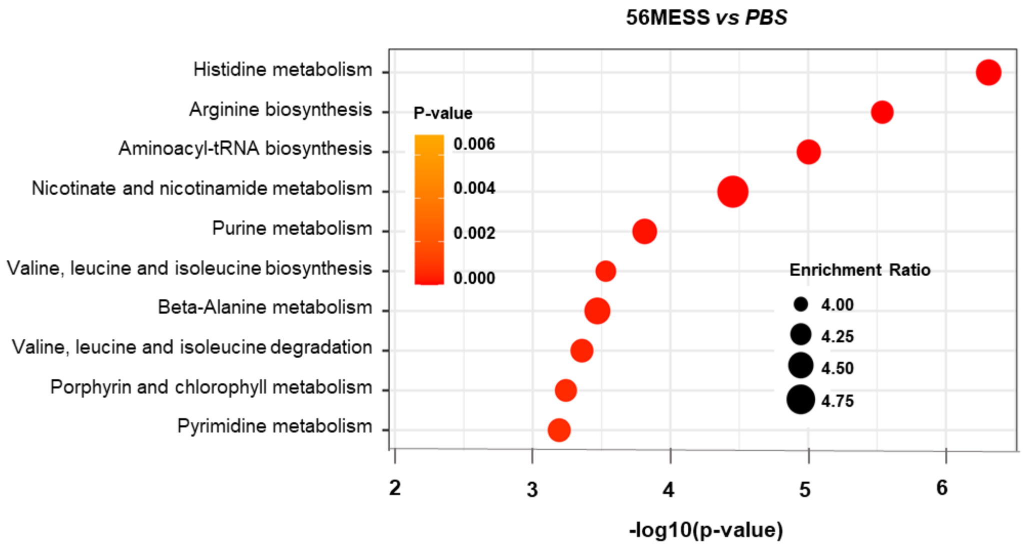

Figure S20. Top ten KEGG annotation of different metabolites between the OCM-1 cells treated with 56MESS and PBS. The size of the dots corresponds to the enrichment ratio, and the colors of the dots correspond to the correlated p-value. (n=3).

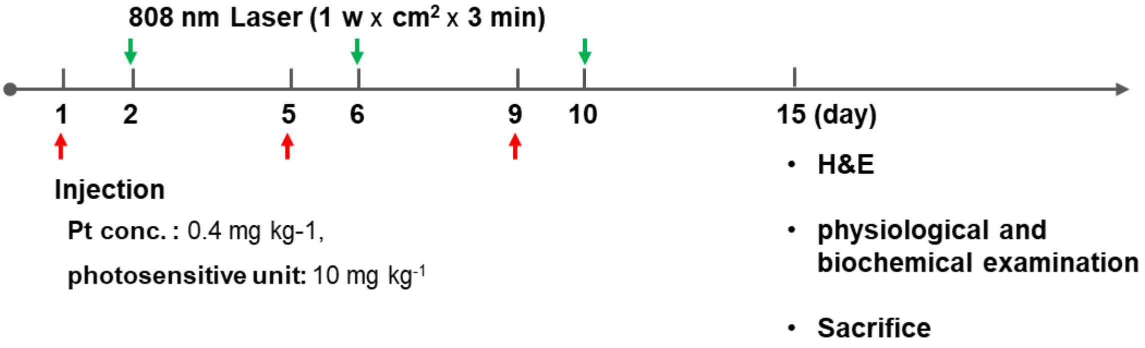

Figure S21. Schematic illustration of the study of biosafety in mice treated with various drugs *in vivo*.

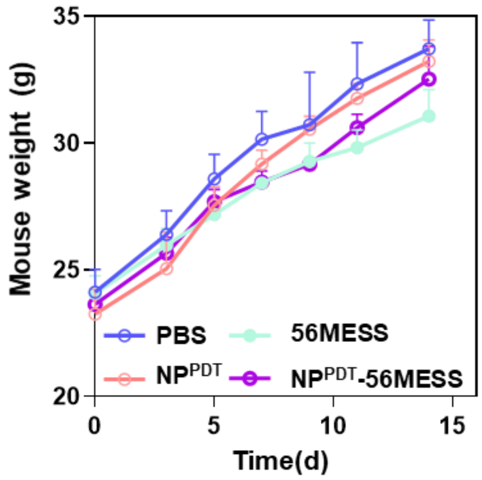

Figure S22. Body weight changes of mice treated with various drugs. 56MESS and NP^{PDT}-56MESS at 0.4 mg Pt/kg body weight. n = 3.

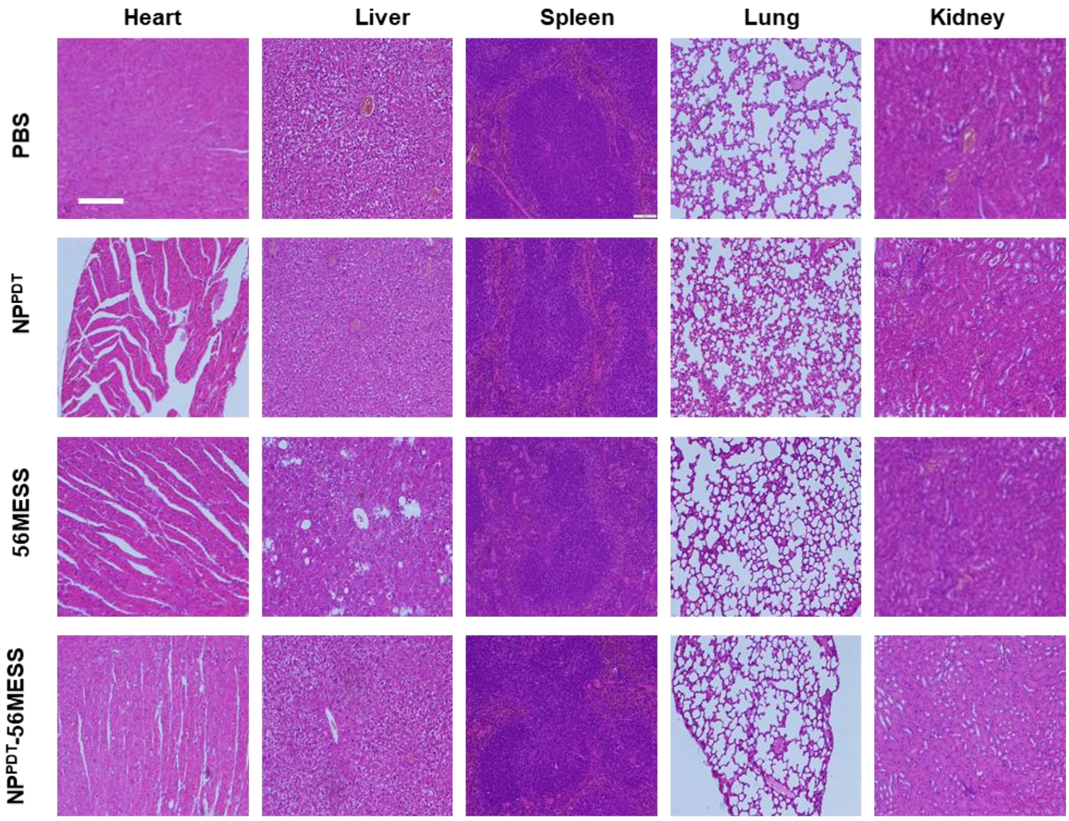

Figure S23. H&E staining of major organs (heart, liver, spleen, lung, and kidney) of KM mice treated with PBS, NP^{PDT}, 56MESS, or NP^{PDT}-56MESS at 0.4 mg Pt/kg body weight. n = 3 mice per group. Scale bar: 200 μm.

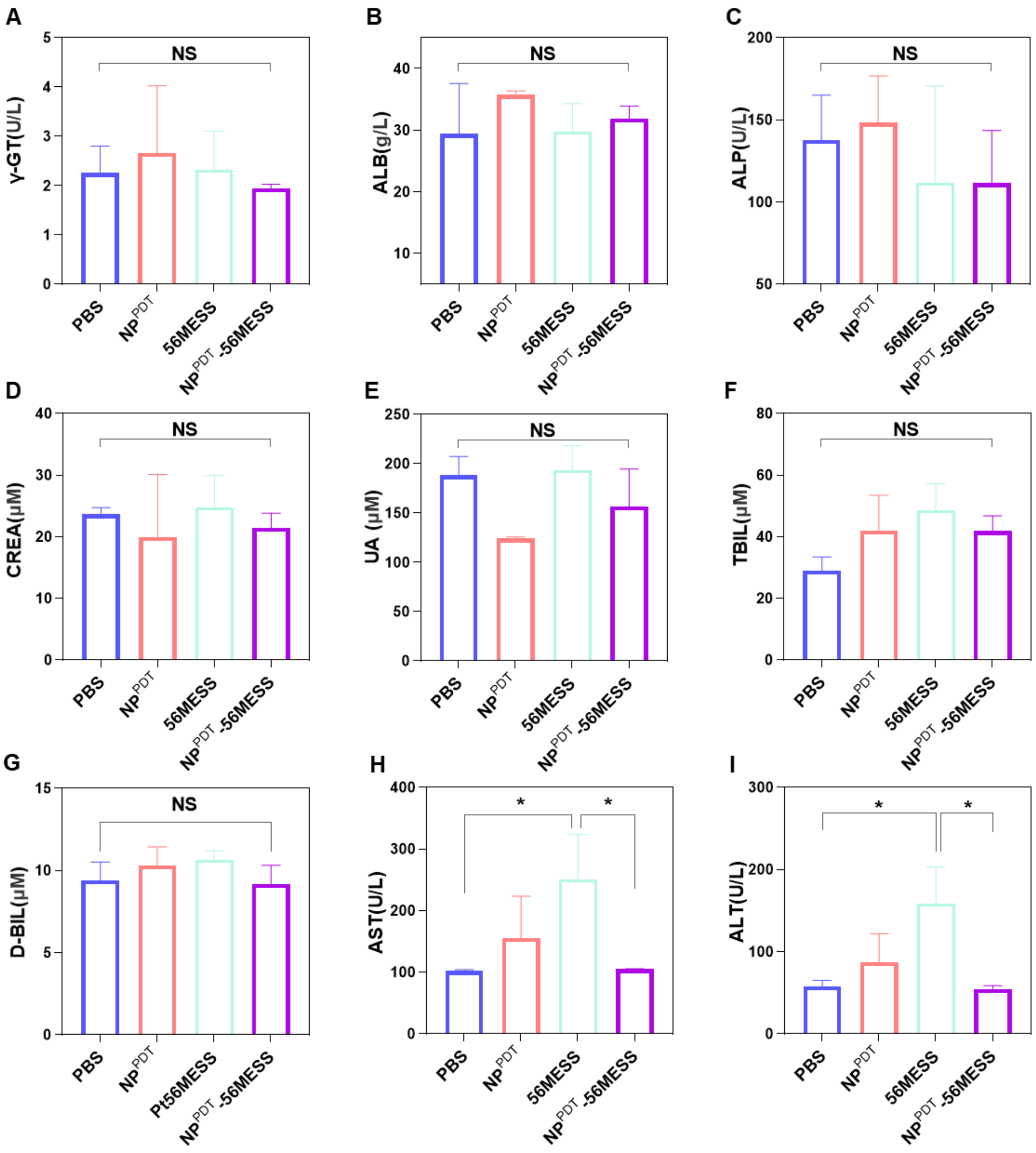

Figure S24. Indexes of liver and kidney function in peripheral blood of KM Mice. (A)-(I) Biochemical analysis of serum of KM mice treated with various drugs: glutamyltransferase (γ-GT), Albumin (ALB), alkaline phosphatase (ALP), creatinine (CREA), uric acid (UA), Total bilirubin (TBIL), direct bilirubinalanine (D-BIL), aspartate aminotransferase (AST), aminotransferase (ALT). n = 3. Data are presented as mean ± SD. Statistical significances between every two groups were calculated *via* one-way ANOVA. *$p < 0.05$.

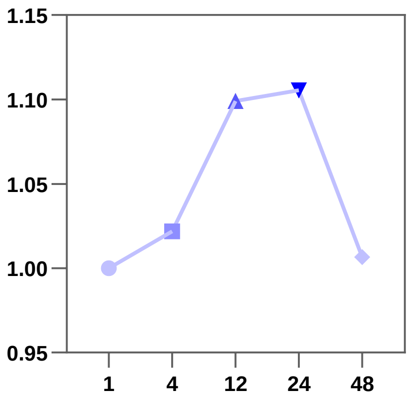

Figure S25. Fluorescence intensity of Cy7.5-labeled NP^{PDT}-56MESS in tumors of OCM-1 bearing mice at different times. n=3

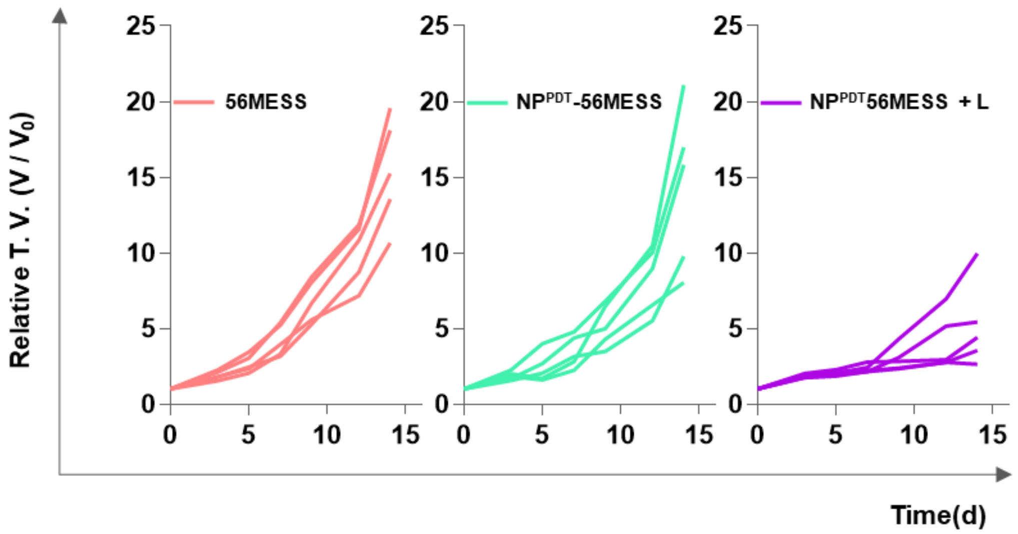

Figure S26. Tumor growth inhibition curves of mice treated with various drugs (Pt at 0.4 mg /kg, n = 5).

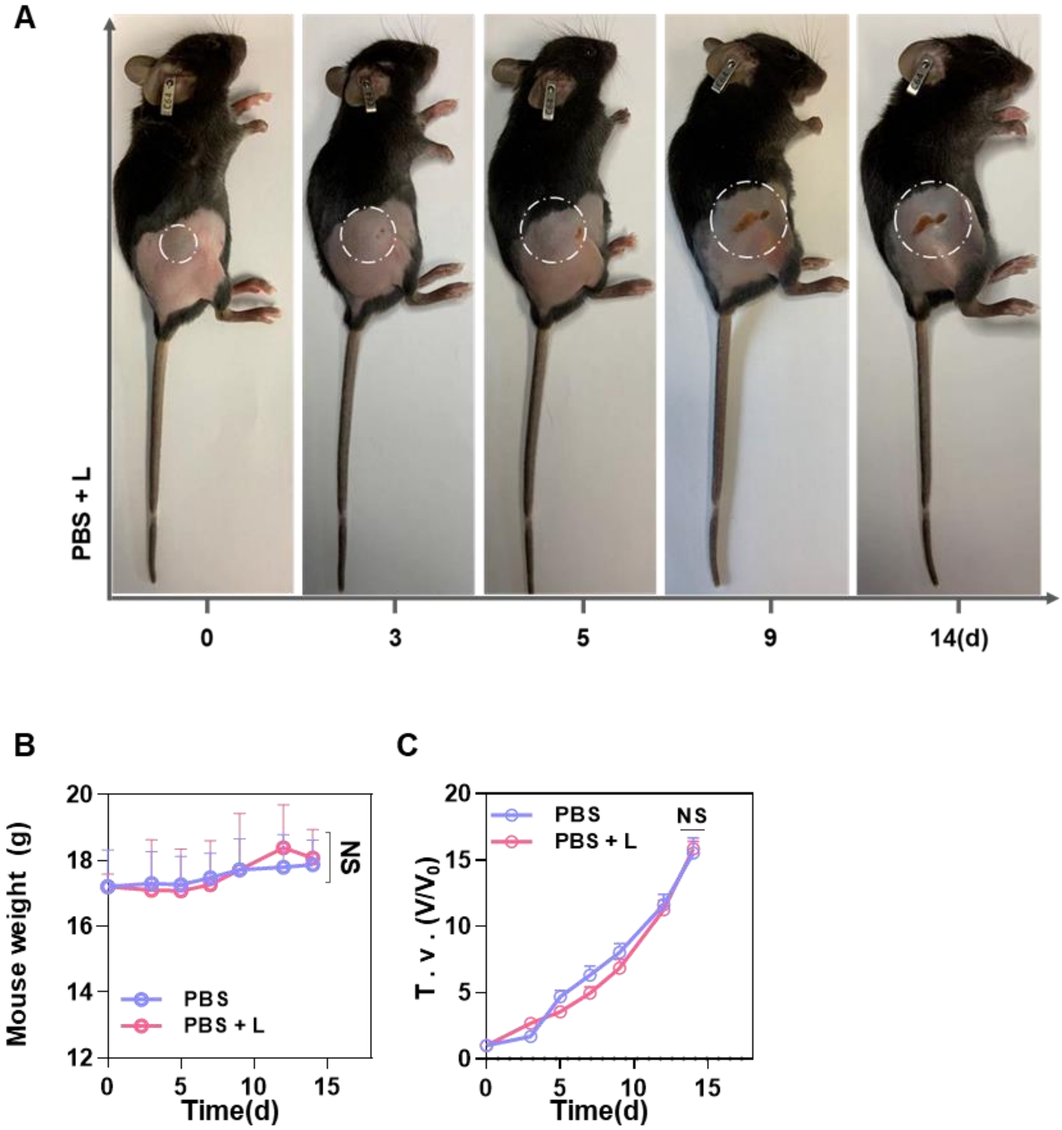

Figure S27. Anti-tumor effect of the PBS + L *in vivo*. (A) Representative images of mice treated with PBS + L at various time points. Body weight changes (B) and tumor growth inhibition curves (C) of mice treated with PBS + L. n = 3. Data are presented as mean ± SD. Statistical significances between every two groups were calculated *via* an unpaired two-sided t-test.

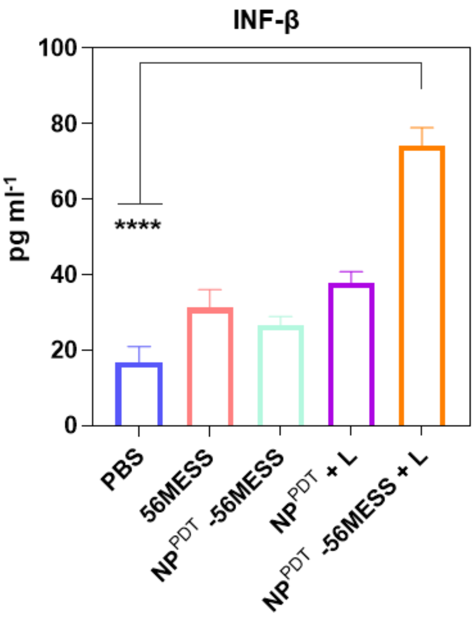

Figure S28. Concentration of IFN- β in mouse serum following various treatments. (Pt at 0.4 mg /kg, n = 5). Data are presented as mean ± SD. Statistical significances between all groups were calculated *via* one-way ANOVA . ****p < 0.0001.

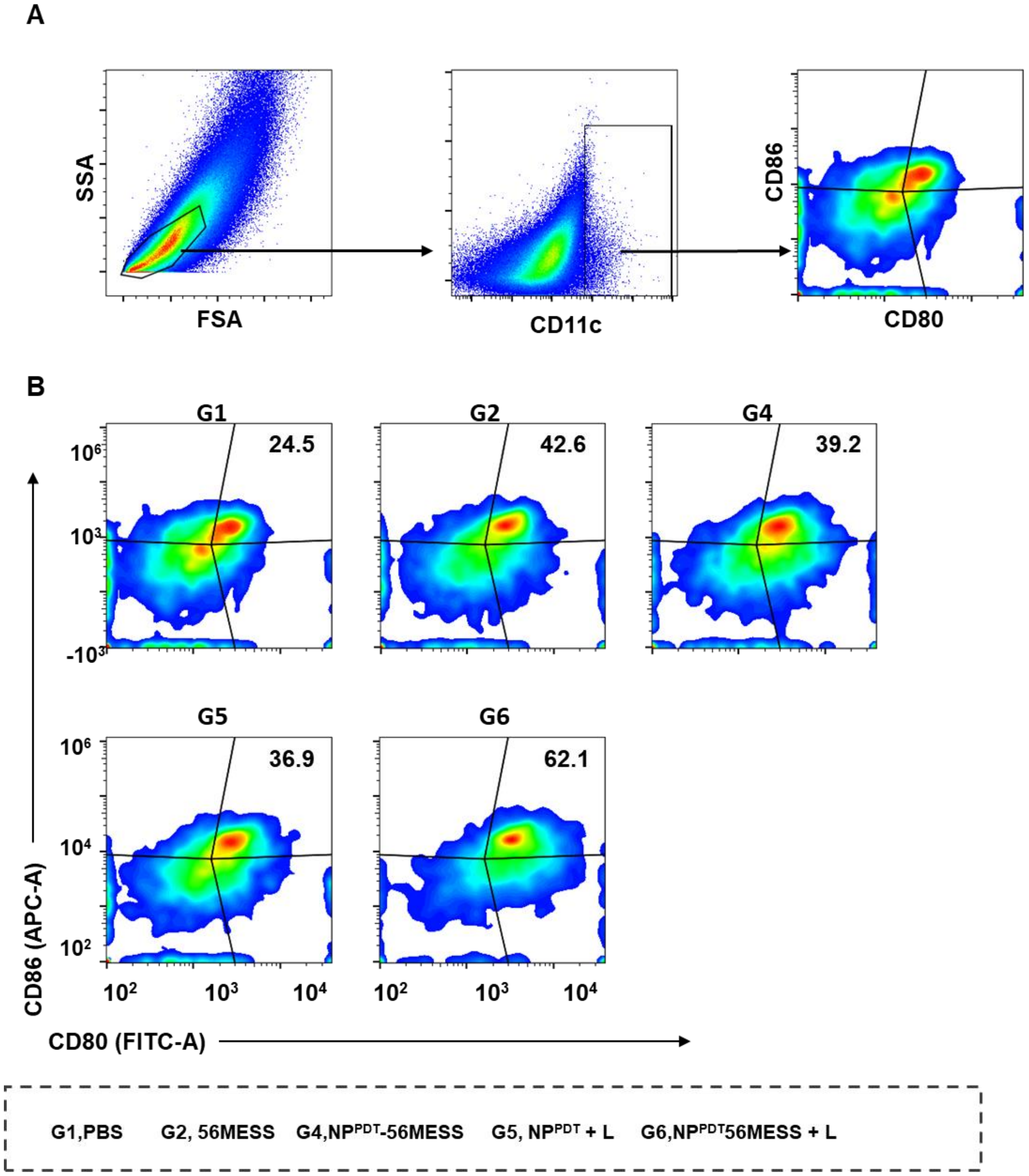

Figure S29. The gating strategy (A) and typical flow cytometer analysis profiles (B) of CD11C$^+$CD80$^+$ CD86$^+$ DCs cells in tumor from B16-F10 tumor bearing mice with different treatments.

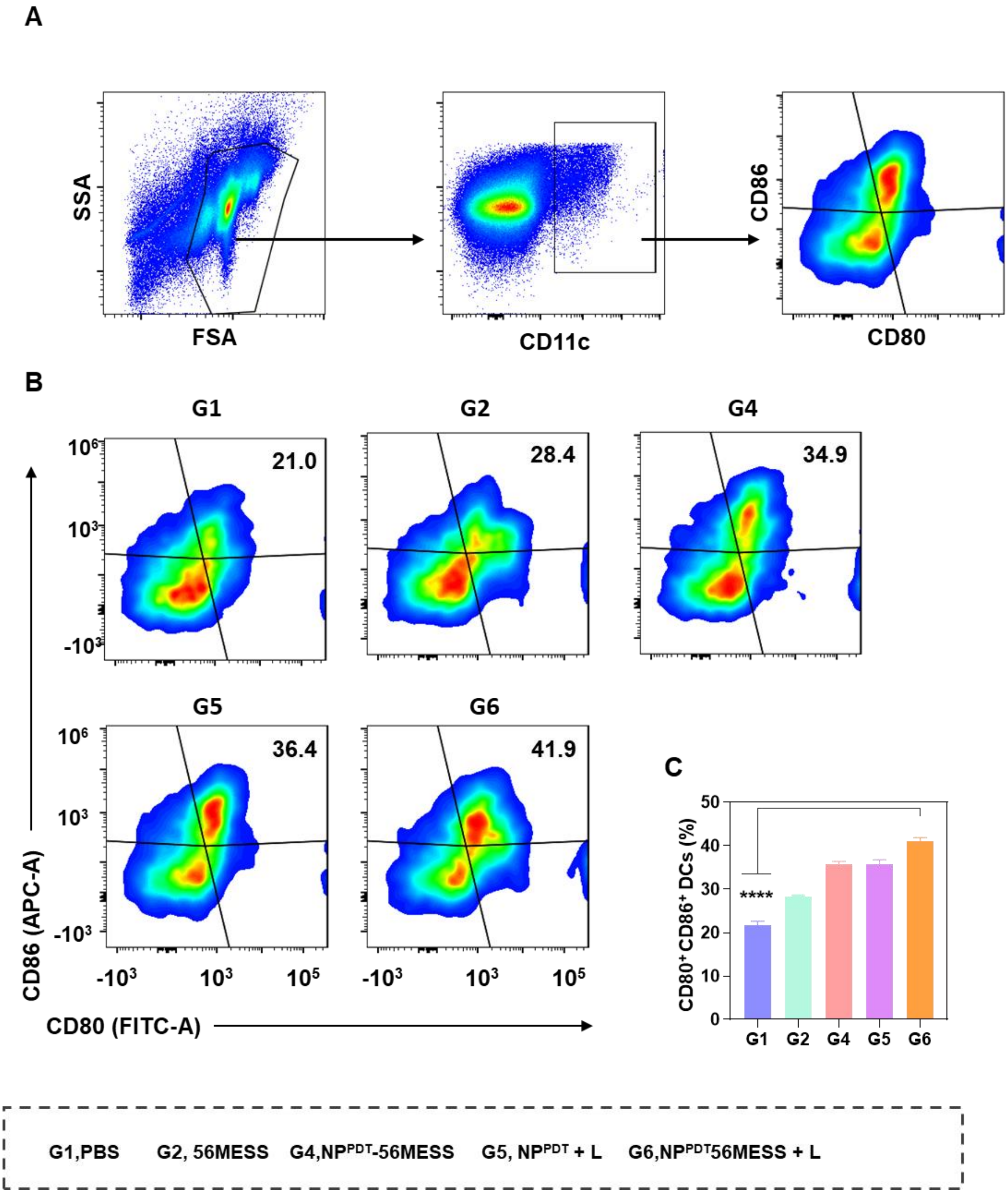

Figure S30. The gating strategy (A) and typical flow cytometer analysis profiles (B) of CD11c$^+$CD80$^+$ CD86$^+$ DCs cells in TDLNs from B16-F10 tumor bearing mice with different treatments. (C) DCs (CD80$^+$ CD86$^+$) populations in TDLNs. n=3. Data are presented as mean ± SD. Statistical significances between all groups were calculated *via* one-way ANOVA . ****p < 0.0001.

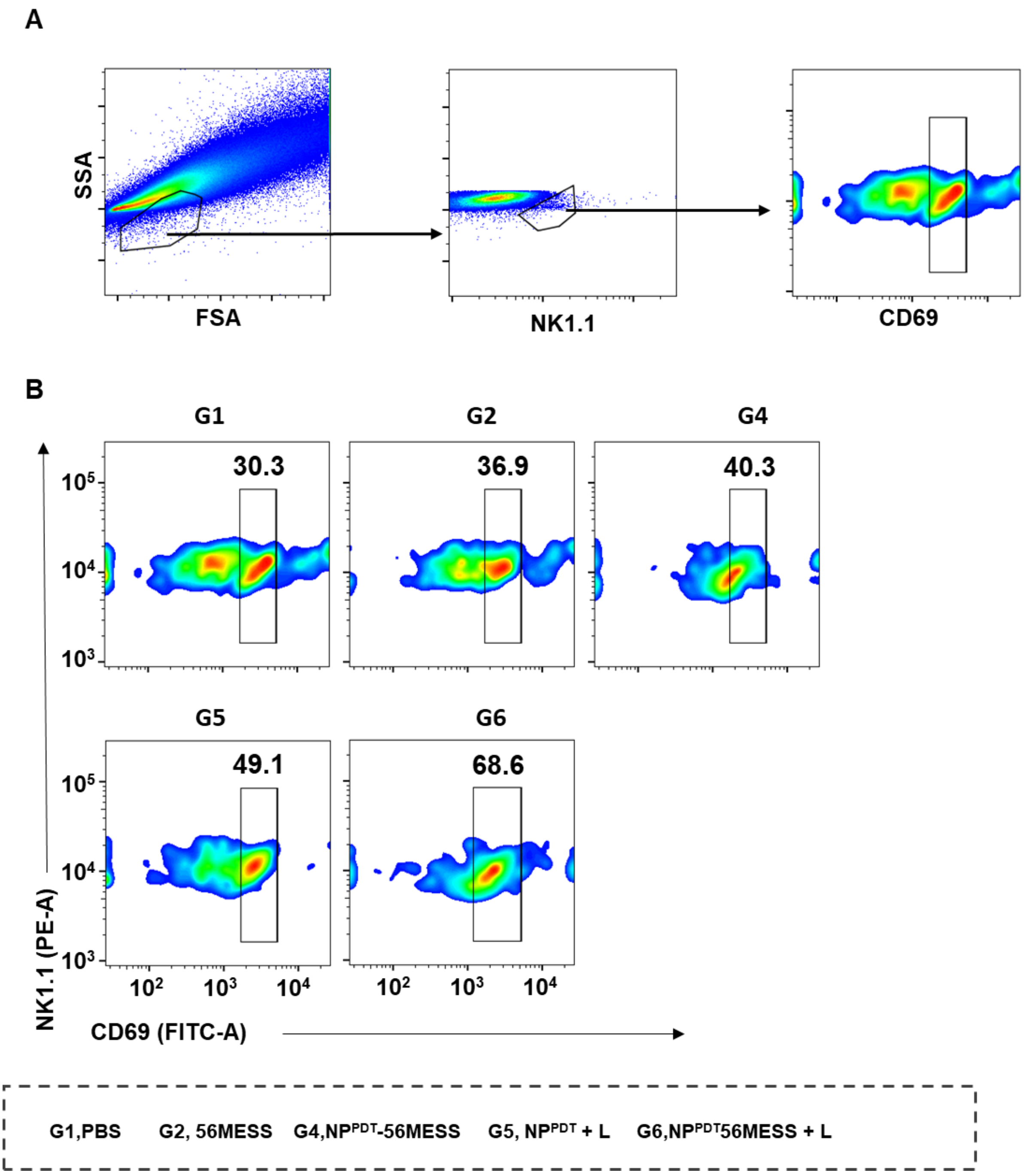

Figure S31. The gating strategy (A) and typical flow cytometer analysis profiles (B) of $CD69^+$ $NK1.1^+$ cells in tumors from B16-F10 tumor bearing mice with different treatments.

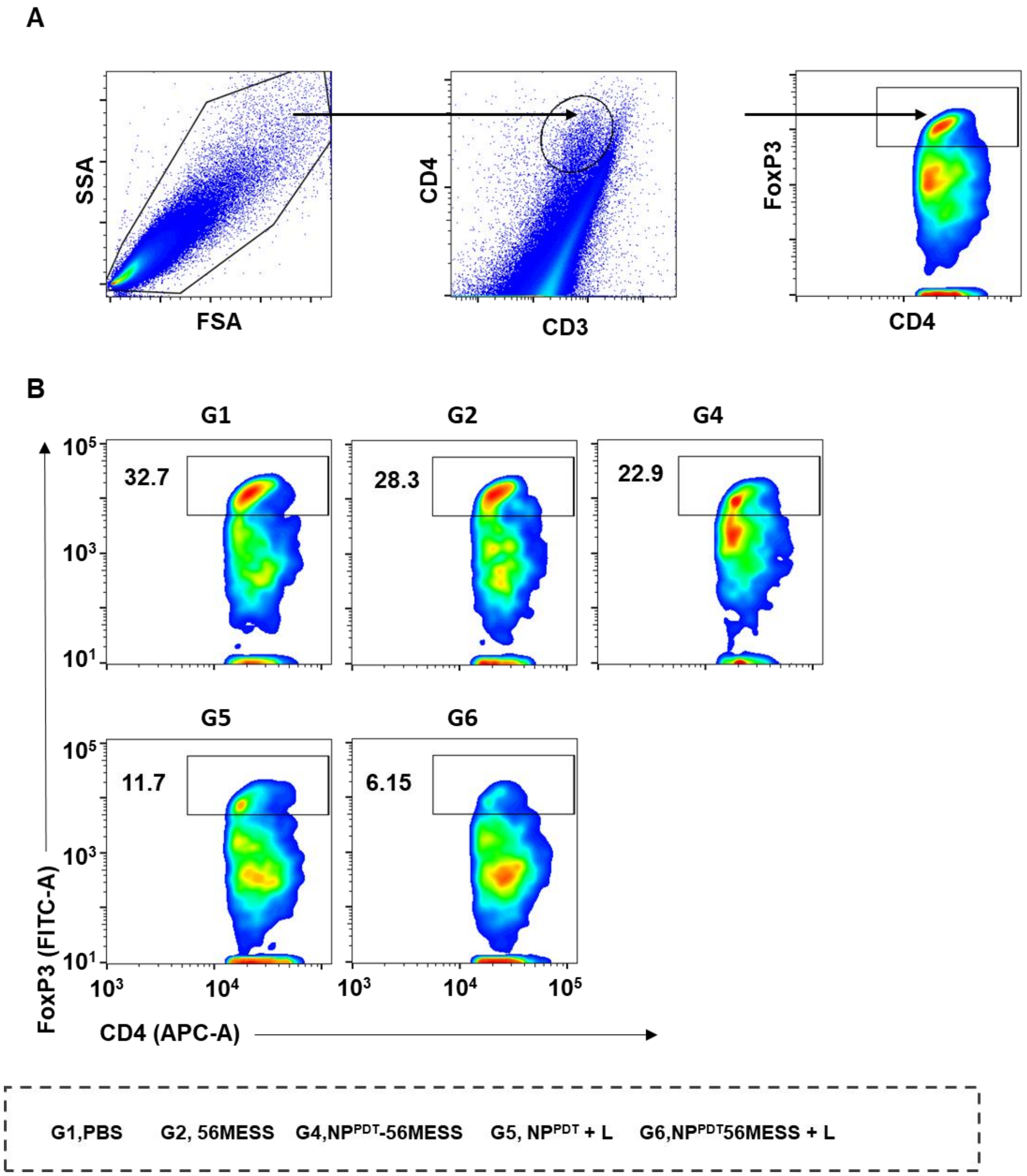

Figure S32. The gating strategy (A) and typical flow cytometer analysis profiles (B) of CD4$^+$ FoxP3$^+$ cells in tumors from B16-F10 tumor bearing mice with different treatments.

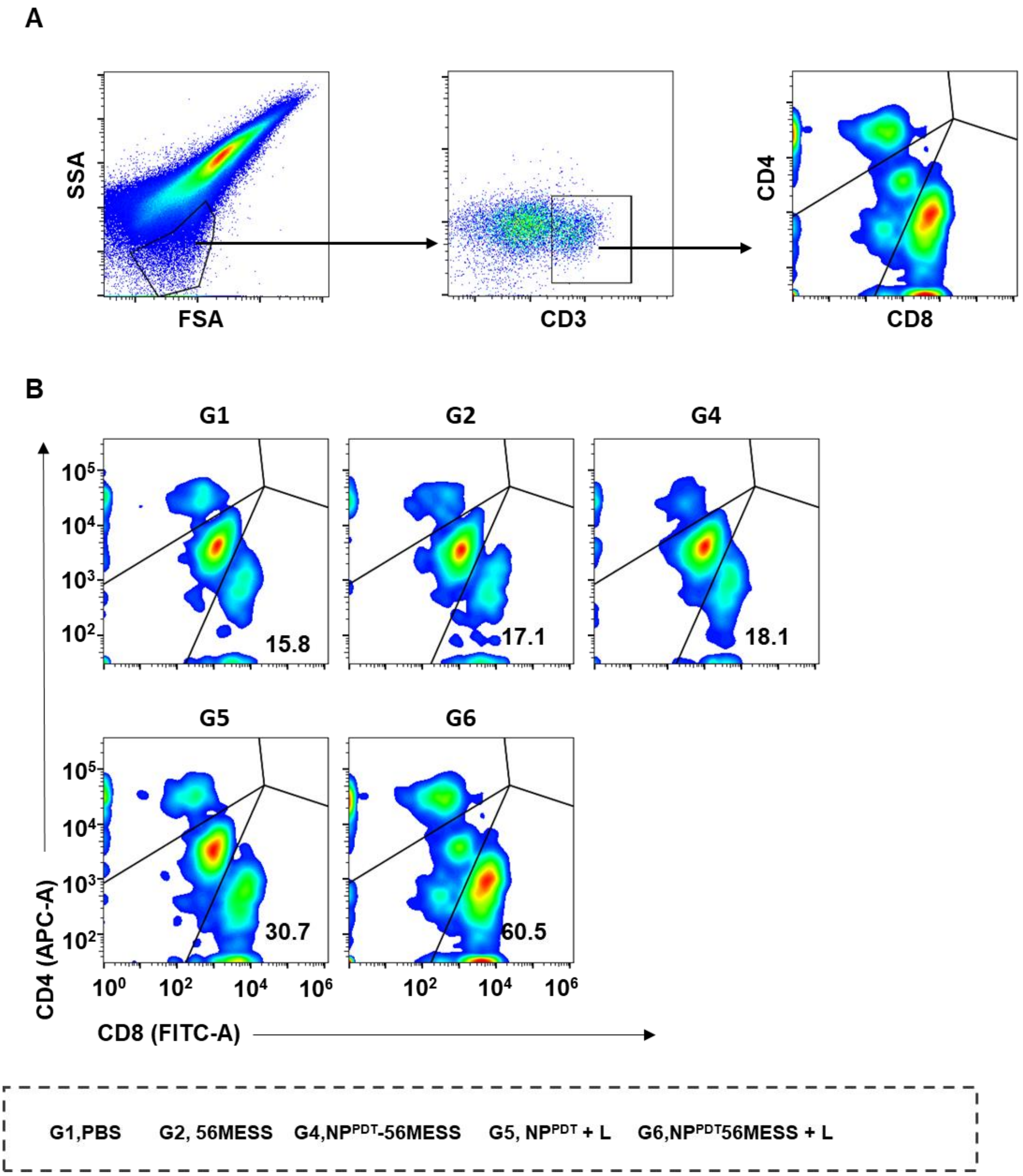

Figure S33. The gating strategy (A) and typical flow cytometer analysis profiles (B) of CD3$^+$ CD8$^+$ or CD3$^+$ CD4$^+$ T cells in tumors from B16-F10 tumor bearing mice with different treatments.

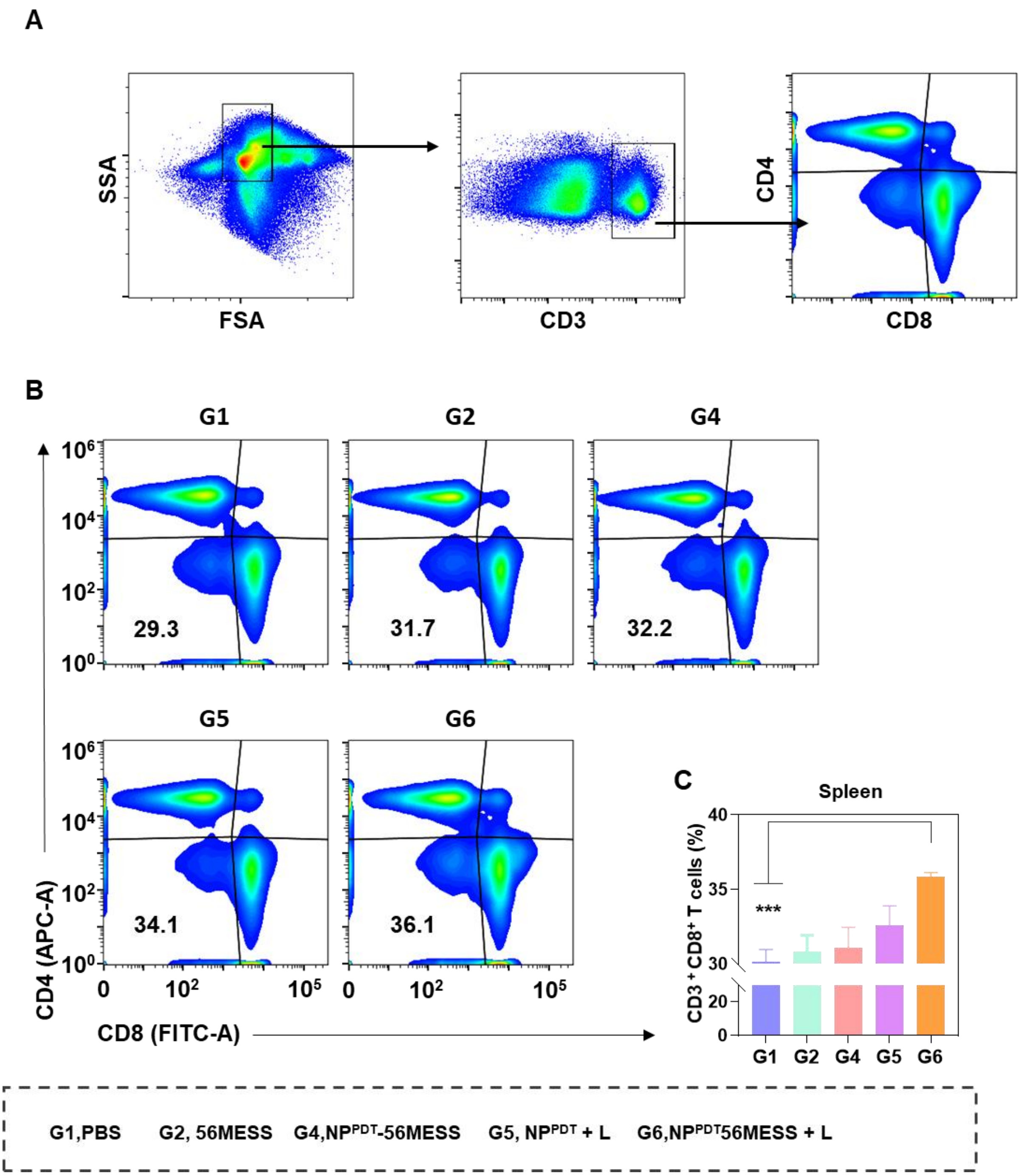

Figure S34. The gating strategy (A) and typical flow cytometer analysis profiles (B) of $CD3^+$ $CD8^+$ or $CD3^+$ $CD4^+$ T cells in spleens from B16-F10 tumor bearing mice with different treatments. (C) $CD8^+$T cells ($CD3^+$ $CD8^+$) populations within spleens. n=3. Data are presented as mean ± SD. Statistical significances between all groups were calculated *via* one-way ANOVA . ****$p < 0.0001$.

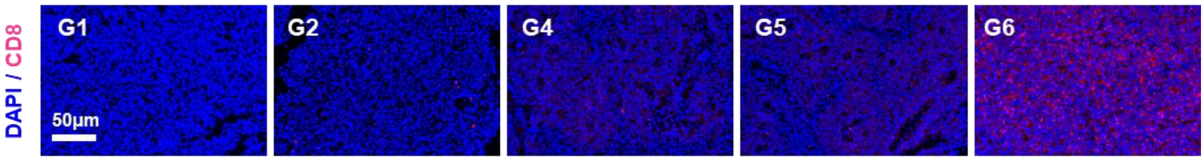

Figure S35. Representative immunofluorescence imaging of the infiltration of $CD8^+$ T cells in B16-F10 tumors upon different treatments. (Blue, DAPI; Red, CD8)

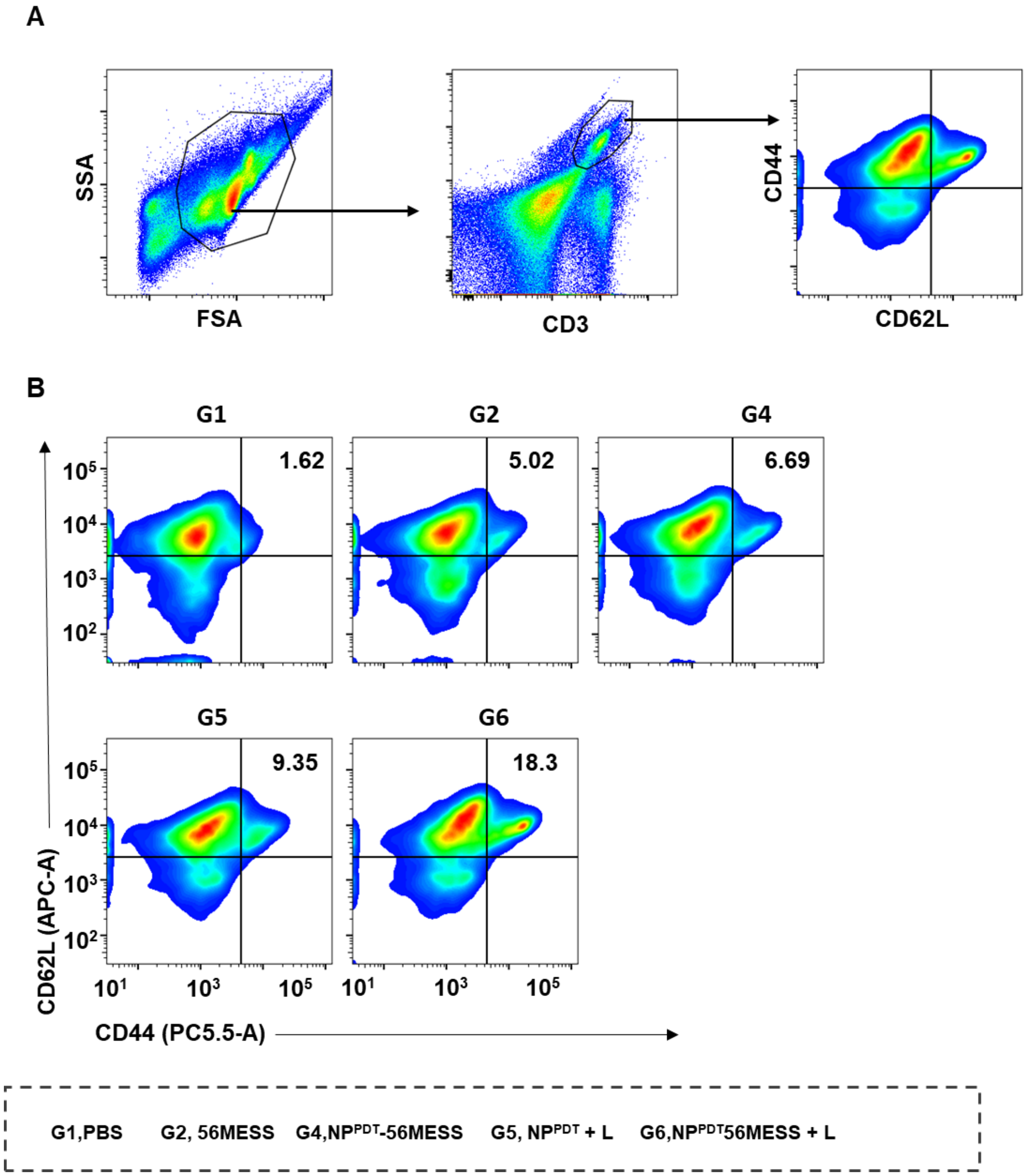

Figure S36. The gating strategy (A) and typical flow cytometer analysis profiles (B) of $CD44^+$ $CD62L^+$T cells in spleens from B16-F10 tumor bearing mice with different treatments.

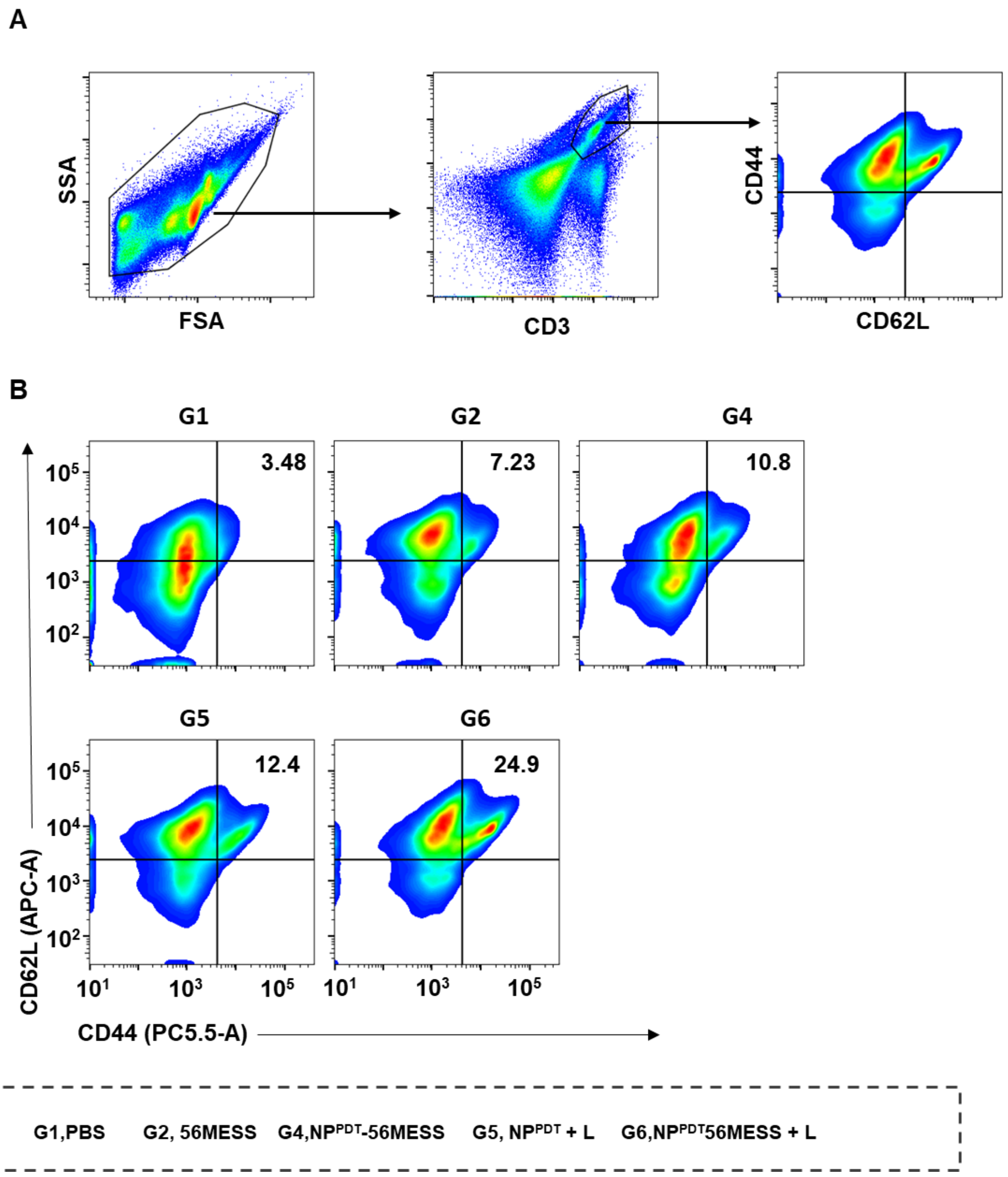

Figure S37. The gating strategy (A) and typical flow cytometer analysis profiles (B) of $CD44^+$ $CD62L^+$T cells in TDLNs from B16-F10 tumor bearing mice with different treatments.

Table S1. Antibodies used for flow cytometry analysis.

Antibody	CompanyCatalog number	Catalog number
Goat polyclonal Secondary Antibody to Rabbit IgG-H&L (Alexa Fluor) ®488	abcam	ab150077
Goat polyclonal Secondary Antibody to Rabbit IgG-H&L (Alexa Fluor) ®555	abcam	ab150078
PE-CD11c	elabscience	E-AB-F0991D
FITC-CD80	elabscience	E-AB-F0992C
APC-CD86	elabscience	E-AB-F1012E
FITC-CD69	elabscience	E-AB-F1187UC
PE-NK1.1	elabscience	E-AB-F0987UH
APC-CD4	elabscience	E-AB-F1097E
FITC-FoxP3	elabscience	E-AB-F1238C
PE-CD3	elabscience	E-AB-F1013D
FITC-CD8	elabscience	E-AB-F1104C
PC5.5-CD44	elabscience	E-AB-1038J
APC-CD62L	Biolegend	E-AB-F1051E

Table S2. Characterization of P1 by gel permeation chromatography (GPC) spectrum.

Polymer	Mn	Mv	MP	Mz	Mz+1	Mz/Mw
P1	16358	18174	20109	19694	20984	1.083685

Mn: Number-average molecular weight; Mw: Weight-average molecular weight; MP: Peak molecular weight; Mz: Size-average molecular weight.

Table S3. Primers used for qPCR quantification of cytosolic mtDNA.

DNA	Forwarde Primer Squence	Reverse Primer Squence
mtDNA	GCTGTCCCCACATTAGGCTT	CGATGGGCATGAAACTGTGG
β-actin	TCACCATGGATGATGATATCGC	AATCCTTCTGACCCATGCC
